# Buried Interface Dielectric Layer Engineering for Highly Efficient and Stable Inverted Perovskite Solar Cells and Modules

**DOI:** 10.1002/advs.202300586

**Published:** 2023-04-25

**Authors:** Huan Li, Guanshui Xie, Xin Wang, Sibo Li, Dongxu Lin, Jun Fang, Daozeng Wang, Weixin Huang, Longbin Qiu

**Affiliations:** ^1^ SUSTech Energy Institute for Carbon Neutrality Department of Mechanical and Energy Engineering Southern University of Science and Technology Shenzhen 518055 China

**Keywords:** aluminum oxide nanoparticles, defect passivation, perovskite solar cells, phenethylammonium bromide, stability

## Abstract

Stability and scalability are essential and urgent requirements for the commercialization of perovskite solar cells (PSCs), which are retarded by the non‐ideal interface leading to non‐radiative recombination and degradation. Extensive efforts are devoted to reducing the defects at the perovskite surface. However, the effects of the buried interface on the degradation and non‐radiative recombination need to be further investigated. Herein, an omnibearing strategy to modify buried and top surfaces of perovskite film to reduce interfacial defects, by incorporating aluminum oxide (Al_2_O_3_) as a dielectric layer and growth scaffolds (buried surface) and phenethylammonium bromide as a passivation layer (buried and top surfaces), is demonstrated. Consequently, the open‐circuit voltage is extensively boosted from 1.02 to 1.14 V with the incorporation of Al_2_O_3_ filling the voids between grains, resulting in dense morphology of buried interface and reduced recombination centers. Finally, the impressive efficiencies of 23.1% (0.1 cm^2^) and 22.4% (1 cm^2^) are achieved with superior stability, which remain 96% (0.1 cm^2^) and 89% (1 cm^2^) of its initial performance after 1200 (0.1 cm^2^) and 2500 h (1 cm^2^) illumination, respectively. The dual modification provides a universal method to reduce interfacial defects, revealing a promising prospect in developing high‐performance PSCs and modules.

## Graphical abstract

The aluminum oxide (Al_2_O_3_) nanoparticles are imbedded into the buried interface, which fills the voids and grain boundaries of perovskite, leading to compact morphology and reduced dangling bonds and defects. The suppressed trap‐assisted recombination, better energy alignment, and decreased *J*–*V* hysteresis in the modified device with Al_2_O_3_ nanoparticles and phenethylammonium bromide contribute to a significant increase in voltage and stability.

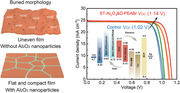

## 1 Introduction

Over the past decades, photovoltaic (PV) technologies as power supply systems have received much attention due to the increased need for energy‐harvesting devices. Among the various techniques, perovskite solar cells (PSCs) are the most popular topic,^[^ ^1^ ^]^ which have seen a significant increase in the power conversion efficiency (PCE) with attractive optoelectronic properties such as adjustable band gap,^[^ ^2^ ^]^ long electron‐hole diffusion length,^[^ ^3^ ^]^ ultrahigh light absorption coefficient,^[^ ^4^ ^]^ and low exciton binding energy.^[^ ^5^ ^]^ To date, the PCE of single‐junction PSCs has skyrocketed to 25.7% from the initial PCE of 3.8%.^[^ ^6^ ^]^ However, most of the PSCs with high PCE are small‐area cells and the PCE decreases remarkably when scaling up, so there is still much room to further improve the performance.^[^ ^7^ ^]^ On the other hand, to approach commercialization, considerable experimental efforts have been concentrated on improving the long‐term stability of PSCs.^[^ ^8^ ^]^ However, the long‐term stability of PSCs is still lagging behind commercial silicon solar cells.^[^ ^8a,8b^ ^]^ Massive defects could be formed at the surface and grain boundaries, resulting in serious non‐radiative recombination which significantly deteriorates the performance of PSCs.^[^ ^9^ ^]^ Therefore, it is important to passivate the defects at the surface and grain boundaries of perovskite film to realize the high efficiency and outstanding stability of PSCs.

So far, a plethora of strategies have been proposed to passivate the defects of perovskite films. The generally used approach for passivating the perovskite top surface is to construct a 2D/3D interface using a solution‐based deposition of organic ammonium salts such as phenylethylammonium,^[^ ^10^ ^]^ butylammonium,^[^ ^11^ ^]^ and oleylammonium salts.^[^ ^12^ ^]^ Specifically, You and co‐workers reported highly efficient PSCs with a certified PCE of 23.32% and an open‐circuit voltage (*V*_OC_) as high as 1.18 V through forming a 2D PEA_2_PbI_4_ perovskite layer above 3D perovskite films using phenethylammonium iodide (PEAI). The defects and non‐radiative recombination were indispensably suppressed.^[^ ^10b^ ^]^ It is well known that the 2D perovskite layer is prepared via a post‐treatment protocol, in which isopropanol (IPA) is applied to dissolve the organic ammonium salts. Nevertheless, IPA is detrimental to the 3D perovskite by dissolving formamidinium iodide (FAI).^[^ ^13^ ^]^ Recently, Mohite's group fabricated phase‐pure 2D halide perovskite with different thicknesses on 3D perovskite by choosing a suitable solvent acetonitrile (MeCN) to dissolve the 2D perovskite precursor, whereas the solubility of 3D perovskite is neglected in MeCN. This method is conducive to protecting the underlying 3D perovskite. A high PCE of 24.5% and remarkable stability of *T*_99_ > 2000 h were achieved.^[^ ^14^ ^]^

In recent years, unremitting efforts have also been made to reduce the buried interfacial defects and improve the carriers' transport beneath the perovskite. For example, the NiO*_x_*/perovskite interface was modified by implementing organic cations with plentiful —NH_2_ groups, for example, guanidinium (GA) cations, leading to a notable increase of 65 mV in *V*_OC_ and a PCE to 22.9%.^[^ ^15^ ^]^ Vaynzof and co‐workers adopted dual interfacial modification to simultaneously improve the *V*_OC_ and fill factor (FF) on the basis of depositing large organic cation PEAI on the top and the buried perovskite film. This protocol is beneficial to grow high‐quality perovskite film, as well as the passivation of the top surface of perovskite film. As a result, maximum *V*_OC_ and FF values of 1.184 V and 85% were reached, respectively.^[^ ^16^ ^]^ Huang et al. have revealed that extensive voids near the buried interface were generated during the deposition process of perovskite, due to the evaporation of residual dimethyl sulfoxide (DMSO) solvent, restricting the PCE and stability of PSCs under illumination.^[^ ^17^ ^]^ Therefore, modifying the buried and top interfaces of perovskite film is imperative for obtaining top‐performing PSCs. Nevertheless, most approaches focused on the top surface of perovskite film. There are scarce works concentrated on solving the bottleneck issues of the buried interface of perovskite film, predominately because the buried interface is not exposed, unlike the top surface of perovskite.^[^ ^18^ ^]^ It is difficult to straightforwardly mitigate the imperfections of the buried interface, leading to a huge room for improvement in the PV performance. Apart from defect passivation and non‐radiation recombination suppression, the buried interface is also vital for the crystal growth process. Among the several works devoted to modifying the buried contact layer, dielectric layer passivation has been applied for high‐performance PSCs, like PMMA^[^ ^19^ ^]^ and polystyrene (PS)^[^ ^20^ ^]^ interfacial layer, but the effect on the underlying dielectric layer is still not clear yet. Researchers have also investigated dielectric inorganics (Al_2_O_3_ and ZrO_2_) to passivate and self‐encapsulate the perovskite film.^[^ ^21^ ^]^ However, it is not widely used due to the sensitivity of electric tunneling to the thickness of the dielectric layer. Furthermore, the synchronous regulation of the buried and top interfaces has rarely been investigated by a combination of the dielectric layer and the organic passivation layer. Hence, rational modification for buried and top interfaces of perovskite film is desperately necessary to reduce undesired charge carrier recombination for high‐performance PSCs.

Here, self‐assembled monolayers (SAMs) were selected as HTL because of their outstanding properties such as easy preparation, minimal required concentration, and scalable fabrication.^[^ ^22^ ^]^ However, it is still challenging to deposit well‐ordered and compact SAMs layer due to the chemical properties of the substrate surface. The presence of imperfections at the substrate reduces the potential binding sites and the absorbability of SAMs on the substrate, and pinholes might be generated in the SAMs which undoubtedly connect the perovskite and bottom electrode, leading to an increment of recombination centers.^[^ ^23^ ^]^ Therefore, in order to reduce the defect densities and non‐radiative recombination at the interfaces of perovskite film, we propose an effective strategy using aluminum oxide (Al_2_O_3_) nanoparticles as a dielectric layer and alkylammonium salt phenethylammonium bromide (PEABr) as a passivation layer. Al_2_O_3_ nanoparticles were introduced into the heterointerface between HTL and perovskite, hampering the direct contact between perovskite and substrate, which result in a 90 mV increase in *V*_OC_ based on [2‐(3,6‐dimethoxy‐9H‐carbazol‐9‐yl)ethyl] phosphonic acid (MeO‐2PACz) HTL and MA‐free perovskite. On the other hand, the effect of PEABr has been studied systematically, which was employed to modify both the buried and top interfaces of perovskite film. It was found that the *V*_OC_ just increased by 20 mV when only PEABr was applied without Al_2_O_3_ nanoparticles. When Al_2_O_3_ nanoparticles and PEABr were simultaneously inserted into the buried perovskite film, and the perovskite top surface was concurrently passivated using PEABr, it is intriguing that 120 mV raise in *V*_OC_ was observed. The significant increase in *V*_OC_ is mainly attributed to the reduced non‐radiative recombination at the buried interface because the Al_2_O_3_ nanoparticles could fill the void between grains and enlarge the grain size, hampering the accumulation of carriers and improving the collection of carriers. The dual modification proposed in this work endows a remarkable increase in PCE from 19.8% to 23.1% based on the precursor composition of Cs_0.15_FA_0.85_Pb(I_0.95_Br_0.05_)_3_. Excitingly, the long‐term and humidity stabilities were also extensively ameliorated. The large‐area device with an area of 1 cm^2^ delivered a PCE as high as 22.4%, showing superior long‐term stability with the remaining 89% PCE of its initial value after continually illuminating for 2500 h. Owing to the scalable property of SAMs, we also fabricated the module based on a 5 cm × 5 cm substrate showing a PCE of 18.7%. The improved PV performance could be mainly attributed to the high‐quality perovskite film, better energy level alignment, faster charge extraction, and reduction of non‐radiative recombination at both the buried and top interfaces. Particularly, the smooth and compact morphology of the buried interface makes a great contribution to improving the PCE and stability. These results prove that this work paves the way for effectively passivating the defects at the buried and top interfaces of perovskite film and reveals the relationship between the morphology of the buried interface and the device performance.

## 2 Results and Discussion

### 2.1 Device Structures

**Figure 1:**
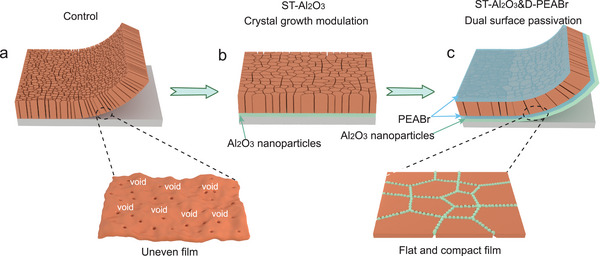
Schematic drawing showing the device structures developed in this work: a) control film structure, b) buried interface treatment of Al_2_O_3_ nanoparticles on the top of HTL (ST‐Al_2_O_3_), and c) the combination of Al_2_O_3_ nanoparticles and PEABr (ST‐Al_2_O_3_& D‐PEABr).

As we know, the bulk defects of perovskite film play an ignorable role with respect to the defects at the surface/interface and grain boundaries.^[^ ^24^ ^]^ Accordingly, dual surface modification was adopted to improve the device's PV performance, and four different kinds of inverted structures were designed, as shown in **Figure** **1** and Figure S1, Supporting Information. The basic device structure (referred to as control, Figure 1a) is ITO/MeO‐2PACz/perovskite/PC_61_BM/C_60_/BCP/Ag prepared by a simple one‐step protocol where ethyl acetate (EA) is used as antisolvent.^[^ ^25^ ^]^ For the purpose of decreasing the defect densities of both interfaces, that is, buried and top surfaces of the perovskite layer, Al_2_O_3_ nanoparticles and PEABr were employed as the interface modification layers. For simplicity, surface treatment using Al_2_O_3_ nanoparticles on MeO‐2PACz is referred to as ST‐Al_2_O_3_ in the following context (Figure 1b), while surface passivation for buried and top sides of perovskite film employing PEABr is referred to as D‐PEABr (Figure S1, Supporting Information). Finally, the dual surface passivated strategies aforementioned are combined, referred to as ST‐Al_2_O_3_&D‐PEABr (Figure 1c). The details of the fabrication process are described in the Experimental Section of Supporting Information.

### 2.2 Photovoltaic Performances

**Figure 2:**
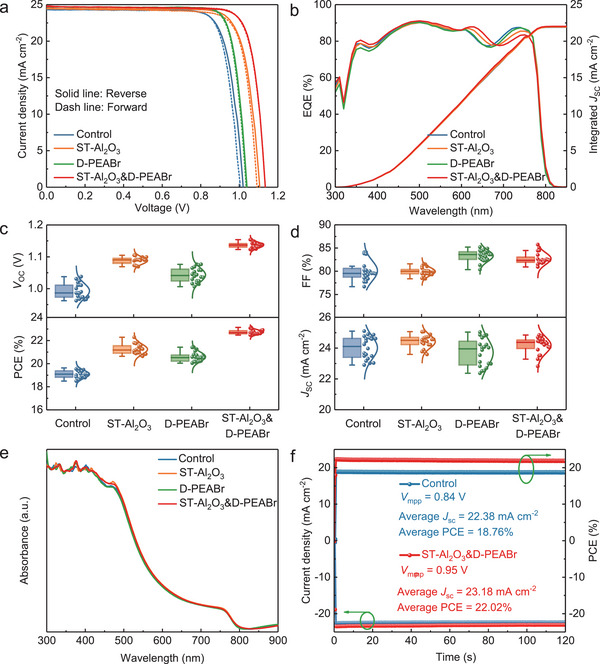
Typical device performance. a) *J*–*V* characteristics, b) EQE spectra, c,d) statistical distribution of the *V*_OC_, FF, *J*_SC_, and PCE of the control, ST‐Al_2_O_3_, D‐PEABr, and ST‐Al_2_O_3_&D‐PEABr devices. e) UV–vis absorption spectra of the perovskite films with various modifications. f) Stabilized power output and the photocurrent density at the initial maximum power point of the control and ST‐Al_2_O_3_&D‐PEABr devices.

**Figure** **2**a exhibits the reverse and forward *J*–*V* curves of the four devices of control, ST‐Al_2_O_3_, D‐PEABr, and ST‐Al_2_O_3_&D‐PEABr. The corresponding PV parameters are summarized in **Table** **1**. It is obvious that the device with the structure of ST‐Al_2_O_3_&D‐PEABr shows the neglected hysteresis behavior, which may be ascribed to the reduced defect densities at the buried and top interfaces of perovskite film, resulting in decreased interfacial charge recombination and suppressed ion migration.^[^ ^26^ ^]^ It is worth noting that the *V*_OC_ is apparently increased by modifying the interfaces. The best control device shows a *V*_OC_ of 1.02 V, a short‐circuit current density (*J*_SC_) of 24.3 mA cm^−2^, and an FF of 80%, leading to a PCE of 19.8%. After the modification using Al_2_O_3_ nanoparticles, the PCE increases to 21.8%, mainly attributed to a significant increase in *V*_OC_, which is enhanced to 1.11 V. The significantly improved *V*_OC_ may attribute to the suppressed trap‐assisted recombination because the incorporation of Al_2_O_3_ fills the grain boundaries and regulates the nucleate and growth process, reducing the contact area of perovskite/HTL and the dangling bonds at the interface, thereby decreasing the interfacial defects, similar to a recent report.^[^ ^27^ ^]^ As for the device of D‐PEABr, the *V*_OC_ shows a slight increase to 1.04 V, while the FF is clearly improved with respect to the control device. Surprisingly, the *V*_OC_ could be further improved to 1.14 V based on the dual modification, which induces an extraordinary improvement in PCE of as high as 23.1% for the best‐performing ST‐Al_2_O_3_&D‐PEABr device. This efficiency is also exciting compared to other MA‐free perovskite devices with inverted structure (Table S1, Supporting Information). To further verify the impact of PEABr and DMF solvent at the buried interface, we prepared three different kinds of devices with and without the passivation of PEABr or DMF washing, and the *J*–*V* curves are illustrated in Figure S2, Supporting Information. The device with the passivation of PEABr at the buried interface illustrates better PV performance than that with only DMF washing or without PEABr passivation, and the DMF washing on the MeO‐2PACz/Al_2_O_3_ film reduces the *J*_SC_. Thus, the treatment of PEABr at the buried interface has a positive effect on the performance of PSCs, rather than DMF. The EQE spectra and the corresponding integrated *J*_SC_ are displayed in Figure 2b. The integrated *J*_SC_ values are 22.25, 22.28, 22.10, and 22.36 mA cm^−2^ for the devices with the structure of control, ST‐Al_2_O_3_, D‐PEABr, and ST‐Al_2_O_3_&D‐PEABr, respectively. The discrepancy of *J*_SC_ determined from EQE spectra and *J*–*V* curves is in the range of 10–20%, which is reasonably precise according to the previous report.^[^ ^28^ ^]^

**Table 1:** Photovoltaic parameters of the perovskite solar cells based on the structure of the control, ST‐Al_2_O_3_, D‐PEABr, and ST‐Al_2_O_3_&D‐PEABr

Device type		*V*_OC_ [V]	*J_S_*_C_ [mA cm^−2^]	FF [%]	PCE [%]
Control	Champion	1.02	24.3	80	19.8
	Average	0.99 ± 0.02	24 ± 0.7	80 ± 2	19.1 ± 0.3
ST‐Al_2_O_3_	Champion	1.11	24.4	80.7	21.8
	Average	1.09 ± 0.01	24.4 ± 0.4	79.9 ± 0.9	21.2 ± 0.4
D‐PEABr	Champion	1.04	24.7	84	21.4
	Average	1.04 ± 0.02	23.7 ± 0.9	83 ± 1	20.6 ± 0.4
ST‐Al_2_O_3_& D‐PEABr	Champion	1.14	24.9	82	23.1
	Average	1.14 ± 0.01	24.2 ± 0.5	83 ± 1	22.7 ± 0.2

The perovskite composition is Cs_0.15_FA_0.85_Pb(I_0.95_Br_0.05_)_3_.

The statistic distributions of *V*_OC_, FF, *J*_SC_, and PCE are exhibited in Figure 2c,d, demonstrating good reproducibility. It can be seen that the *J*_SC_ hardly changes upon surface treatments of Al_2_O_3_ nanoparticles and PEABr, indicating that the light absorption is not affected which is consistent with the results of UV–vis absorption and transmission spectra (Figure 2e and Figure S3a, Supporting Information). And the optical bandgap of ≈1.57 eV was calculated from Tauc plots, which is slightly impacted by different treatments at the interface (Figure S3b, Supporting Information). The improvement of *V*_OC_ is not completely associated with the expansion of the bandgap because the increase in the bandgap is marginal. It probably is related to the improved quality of perovskite film, increased grain size, and decreased charge accumulation which will be elaborated in detail in the following. Figure 2f displays the stabilized power output and the photocurrent density at the initial maximum power point (MPP) of the control and ST‐Al_2_O_3_&D‐PEABr devices. Under continuous illumination, the stabilized PCE of the ST‐Al_2_O_3_&D‐PEABr device is much beyond the control. It can be concluded that the improvement in *V*_OC_ is supreme for ST‐Al_2_O_3_&D‐PEABr devices among the four kinds of PSCs, manifesting the importance of simultaneous modifications of perovskite/MeO‐2PACz and perovskite/PCBM interfaces.

### 2.3 Morphology Improvement and Better Energy Level Alignment

**Figure 3:**
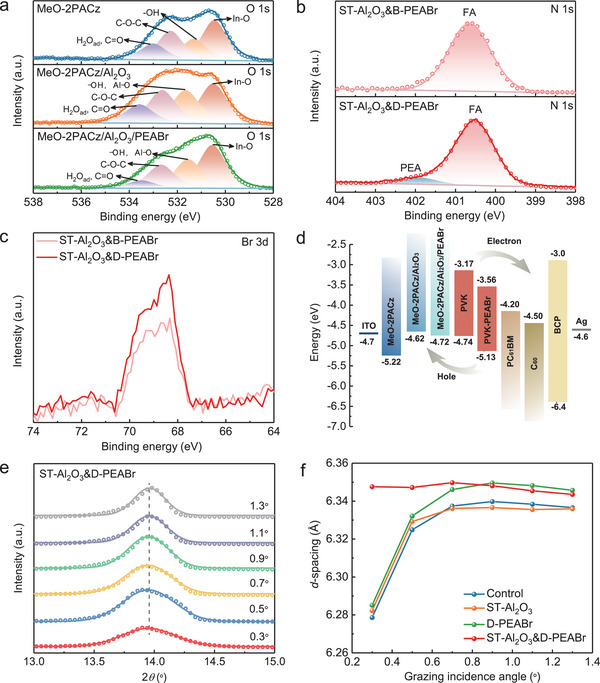
a) XPS spectra of the O 1s for MeO‐2PACz, MeO‐2PACz/Al_2_O_3_, and MeO‐2PACz/Al_2_O_3_/PEABr films. XPS spectra of b) N 1s and c) Br 3d for ST‐Al_2_O_3_&B‐PEABr and ST‐Al_2_O_3_&D‐PEABr perovskite films, in which ST‐Al_2_O_3_&B‐PEABr denotes the perovskite film without the top passivation of PEABr and other passivation layers are same as the ST‐Al_2_O_3_&D‐PEABr perovskite film. d) Schematic energy level alignment diagram of individual layers in the device. e) GIXRD patterns of ST‐Al_2_O_3_&D‐PEABr film at the buried interface with different incidence angles (*α*). f) *d*‐spacing of the (100) crystal plane of perovskite films with different surface treatments.

In order to have a deep insight into the enhancement of *V*_OC_, the X‐ray photoemission spectra (XPS) measurement was performed to study the surface chemical properties. The XPS survey spectra of MeO‐2PACz, MeO‐2PACz/Al_2_O_3_, and MeO‐2PACz/Al_2_O_3_/PEABr films are shown in Figure S4, Supporting Information. The peaks of C, N, O, P, Sn, and In are observed for all films, while peaks arising from Al are just found for MeO‐2PACz/Al_2_O_3_ and MeO‐2PACz/Al_2_O_3_/PEABr films, indicating the presence of Al_2_O_3_ nanoparticles on the MeO‐2PACz surface, as evidenced by the XPS spectra of O 1s core level (**Figure** **3**a). The calibration of XPS spectra for MeO‐2PACz, MeO‐2PACz/Al_2_O_3_, and MeO‐2PACz/Al_2_O_3_/PEABr films was based on the In peak from the ITO substrate.^[^ ^29^ ^]^ As for the MeO‐2PACz film (Figure 3a), the largest peak at 530.5 eV should correspond to the oxygen of In_2_O_3_ originating from the ITO substrate, and the peak at 531.3 eV can be assigned to hydroxyl species (—OH) origination from MeO‐2PACz and the surface of In_2_O_3_ which is important to the absorption of MeO‐2PACz on the substrate.^[^ ^30^ ^]^ Additionally, we find the contribution of C—O—C belonging to the methoxy groups of MeO‐2PACz in accordance with the previous study.^[^ ^31^ ^]^ The peak at the highest binding energy may originate from other hydroxides and adventitious contaminants.^[^ ^30^, ^31^ ^]^ We speculate that P—O species could not be resolved here because of limited sensitivity and possible overlap with the contaminant peaks. It should be mentioned that the O 1s peak shapes of MeO‐2PACz/Al_2_O_3_ and MeO‐2PACz/Al_2_O_3_/PEABr films are various from MeO‐2PACz film, and the peak of —OH deviate to higher binding energies compared with MeO‐2PACz film, which may be related to the introduction of Al_2_O_3_. Since the peak of the Al—O bond in Al_2_O_3_ is located at 531.5 eV,^[^ ^21^, ^32^ ^]^ which is very close to the peak of —OH (531.3 eV). Additionally, the EDX element mapping and line scan also illustrate that the Al_2_O_3_ nanoparticles can be spin‐coated onto the surface of the MeO‐2PACz film (Figure S5a–d, Supporting Information). The XPS spectra of C 1s are apparently deconvoluted into four peaks of C—C, C—N, C—O—C, and C—P (Figure S6a, Supporting Information), where C—N and C—P are related to the carbazole group in MeO‐2PACz, indicating that the MeO‐2PACz is strongly absorbed onto ITO substrate.^[^ ^30^, ^33^ ^]^ The obvious shift of C 1s core level to higher binding energy is found after the deposition of Al_2_O_3_ on the MeO‐2PACz substrate, which shows the discernible changes of the chemical environment of C element, probably indicative of the interaction between Al_2_O_3_ and MeO‐2PACz. Figure S6b,c, Supporting Information, further confirms the occurrence of N and P elements in XPS spectra, where the N element originates from MeO‐2PACz and PEABr, P element comes from MeO‐2PACz.

To study the top surface chemistry of perovskite film with and without passivation of PEABr, we fabricated ST‐Al_2_O_3_&B‐PEABr and ST‐Al_2_O_3_&D‐PEABr perovskite films, where ST‐Al_2_O_3_&B‐PEABr denotes the perovskite film without the top surface passivation of PEABr and other passivation layers are same as the ST‐Al_2_O_3_&D‐PEABr perovskite film. Figure 3b,c displays the XPS spectra of N 1s and Br 3d. A new N 1s peak from PEA^+^ is observed in the ST‐Al_2_O_3_&D‐PEABr film, revealing that the PEABr exists on the top surface of the perovskite layer. The XPS spectra of C 1s, Pd 4f, I 3d, and Cs 3d core levels are presented in Figure S7a–d, Supporting Information. The C 1s spectra are separated into four peaks of C—C (≈284.6 eV), C—N (≈286 eV), N^+^=CH—N (≈288 eV), and C—O/C=O (≈286 to 289 eV), where C—C and C—N bonds belong to the FA^+^ and PEA^+^ of perovskite film, and the N^+^=CH—N and C—O/C=O are assigned to FA^+^ and oxygen/moisture, respectively.^[^ ^10^, ^34^ ^]^ A slight shift of Pb 4f is detected, which is attributed to the interaction between the Pb^2+^ and —NH_2_ group of PEABr, indicating the effective passivation of the perovskite surface, and Br/Pb atomic ratio of ST‐Al_2_O_3_&D‐PEABr film increases compared to that of ST‐Al_2_O_3_&B‐PEABr film, suggesting that more Br^−^ anions are accumulated at the perovskite surface. Besides, the ultraviolet photoelectron spectroscopy (UPS) measurement was conducted to explore the impact of surface treatments on the energy level alignment between individual layers. Figure S8a–c, Supporting Information, shows the second electron cutoff edge and the valence band maximum (VBM) with respect to the Fermi level of the films with different modifications, which can be used to calculate the work function. As shown in Figure S8d, Supporting Information, the Fermi level of perovskite film with top surface passivation of PEABr shifts toward conduction band minimum (CBM), inducing a more n‐type property benefiting the extraction of electrons. Subsequently, the schematic energy level alignment diagram of individual layers in the device is displayed in Figure 3d, where the energy levels of ITO, PC_61_BM, C_60_, BCP, and Ag are obtained from references.^[^ ^35^ ^]^ It is found that the synergistic effect of surface modifications using Al_2_O_3_ nanoparticles and PEABr at the buried interface results in a better energy level alignment, reducing the energy loss. Meanwhile, the energy level offset between perovskite and PCBM decreases after the PEABr treatment, which may be attributed to the formation of dipole moments between Br anions and PEA cations. Compared to PEAI passivation, a larger energy shift could be expected for PEABr because of the higher electronegativity of Br^−^ than I^−^, which leads to a more appropriate energy level alignment for transporting electrons, similar to previous reports.^[^ ^36^ ^]^ The UPS results account for a significant improvement in *V*_OC_, which is in good agreement with the PV performance discussed before.

Grazing incidence X‐ray diffraction (GIXRD) was performed to investigate the crystallinity and residual strain of perovskite films. Figure S9a, Supporting Information, exhibits the GIXRD patterns of perovskite films for buried and top interfaces with various treatments at the grazing incidence angle (*α*) of 1°. For measuring the perovskite film of the buried interface, the perovskite film was peeled off from the device encapsulated by UV‐curing glue and glass. It can be seen that an extra diffraction peak of perovskite film at the buried side appears with respect to the top perovskite film. The new peak could be assigned to the (003) plane of PbI_2_,^[^ ^25^ ^]^ and the area ratio of perovskite (100) to PbI_2_ (003) is calculated, as shown in Figure S9b, Supporting Information. The increased area ratio in ST‐Al_2_O_3_&D‐PEABr film suggests a superior crystal quality of perovskite film, probably owing to decreased defect states and improved morphology at the buried interface of perovskite film. According to previous reports,^[^ ^37^ ^]^ the 2D perovskite phase might be formed on the 3D perovskite surface after the passivation of organic ammonium salts on the top surface of the perovskite film. However, we do not observe the peaks of the 2D phase for D‐PEABr and ST‐Al_2_O_3_&D‐PEABr films. When decreasing the *α* to 0.5° and 0.3° (Figure S9c, Supporting Information), we still not detected the signals of the 2D phase. Note that the penetration depth of X‐ray decreases with decreasing *α*, meaning that the surface structure could be collected with a small *α*. For ST‐Al_2_O_3_&D‐PEABr perovskite film, the diffraction peaks of the 2D phase denoted by black diamonds and heart‐shaped symbol start to emerge as increasing the concentration of PEABr to 5 mg mL^−1^ (Figure S9d, Supporting Information). Thus, we could conclude that the PEABr on the top surface of perovskite film serves as an effective and simple passivation layer, rather than the 2D perovskite phase.^[^ ^10b^ ^]^ The possible reason is that the concentration of PEABr in our work is too low to form a 2D phase. In addition, the residual strain of the buried interface of perovskite films with variable surface treatments is evaluated from GIXRD patterns collected at different *α* (from 0.3° to 1.3°), as shown in Figure 3e and Figure S10, Supporting Information. It is clear that different degree of residual strain exists in the perovskite films at the buried interface with and without modifications. Figure 3f shows the (100) lattice spacing of buried perovskite film changes with an increase of *α*. For control, ST‐Al_2_O_3_, and D‐PEABr films, the position of the (100) plane shifts to lower angles as increasing the *α*, corresponding to the expansion of lattice spacing. However, the (100) lattice spacing of ST‐Al_2_O_3_&D‐PEABr film hardly changes, suggesting that the residual stress is released which is conducive to improving the performance of the device, in consistence with XPS and UPS results.

**Figure 4:**
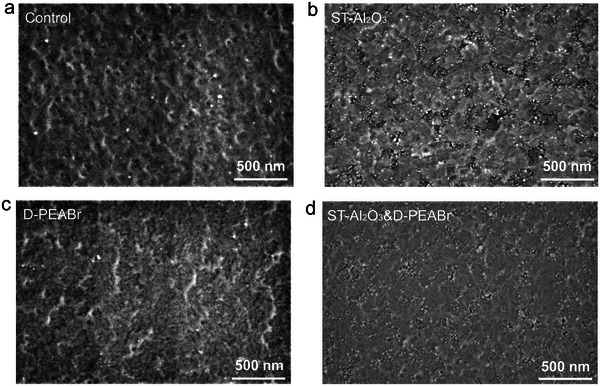
SEM images at the buried interface of a) control, b) ST‐Al_2_O_3_, c) D‐PEABr, and d) ST‐Al_2_O_3_&D‐PEABr perovskite films.

The morphologies of the buried and top interfaces of perovskite films were investigated by the scanning electronic microscope (SEM) and the atomic force microscope (AFM). The top‐view SEM, cross‐section SEM, and the crystal size distributions of control, ST‐Al_2_O_3_, D‐PEABr, and ST‐Al_2_O_3_&D‐PEABr perovskite films are shown in Figures S11 and S12, Supporting Information. It is clear that all perovskite films illustrate superior morphology with compact coverage and pinhole‐free structure. From the cross‐section SEM images, the Al_2_O_3_ nanoparticles can be observed in ST‐Al_2_O_3_ and ST‐Al_2_O_3_&D‐PEABr films. The grain size distribution suggests that the mean grain sizes of ST‐Al_2_O_3_, D‐PEABr, and ST‐Al_2_O_3_&D‐PEABr films increase slightly compared with the control film, probably attributing to the enhanced contact angle (Figure S13a–d, Supporting Information).^[^ ^38^ ^]^ We speculate that the increased contact angle could ameliorate the moisture and operational stabilities. An increase in grain size leads to decreased grain boundaries, enhanced crystallinity, and charge transport, which alleviates the undesirable charge recombination.^[^ ^39^ ^]^ Furthermore, the film with the modification of Al_2_O_3_ has larger surface roughness, as shown in Figure S14a–d, Supporting Information. The root‐mean‐square (RMS) values of MeO‐2PACz/Al_2_O_3_ and MeO‐2PACz/Al_2_O_3_/PEABr films were determined to be 42.8 and 46 nm, respectively, compared to the MeO‐2PACz (3.67 nm) and MeO‐2PACz/PEABr (3.98 nm) films. The increased surface roughness is attributed to the coverage of Al_2_O_3_ nanoparticles with a size of around 15–50 nm on the MeO‐2PACz substrate. The increased roughness will enlarge the crystal grains and reduce the voids in‐between perovskite grains, which decreases the surface recombination centers, thereby suppressing the non‐radiative recombination at the interface. On the other hand, the hole carries can transport through the space between Al_2_O_3_ nanoparticle which is infilled by perovskite. Both positive effects are beneficial to improve the photoelectric performance of devices. For ST‐Al_2_O_3_&D‐PEABr perovskite film, the RMS value decreases to 7.84 nm, compared to the ST‐Al_2_O_3_&B‐PEABr perovskite film (8.57 nm) (Figure S15a,b, Supporting Information), which may be ascribed to the filled grain boundaries, in favor of reducing the defects at the perovskite/PCBM interface. **Figure** **4**a–d shows the buried SEM images of perovskite films with different treatments. It can be seen that the control and D‐PEABr films (Figure 4a,c) are uneven and some voids can be observed at the buried interface. However, the ST‐Al_2_O_3_ and ST‐Al_2_O_3_&D‐PEABr films are flat and compact (Figure 4b,d). Compared to ST‐Al_2_O_3_, fewer voids are detected in the ST‐Al_2_O_3_&D‐PEABr film, inducing fewer degradation paths and improved stability. It is believed that the charge recombination mainly happens in the grain boundaries and interface, due to the dangling bonds in the surface termination groups, and non‐compact contact between the perovskite layer and the bottom layer, which is expected to show remarkable exposure dangling bonds as recombination centers, impacting the collection of charged carriers, thereby resulting in the accumulation of charge carriers and degradation of perovskite films or devices.^[^ ^40^ ^]^ In our case, the Al_2_O_3_ nanoparticles could be imbedded into the buried interface, which enlarges grain size, and fills the voids at the interface of perovskite/HTL and grain boundaries, leading to the packed morphology. The dense morphology could reduce the dangling bonds, the recombination centers, and defects at the interface, causing significant suppressed trap‐assisted recombination. Meanwhile, the ion migration channels could also be dwindled because of decreased imperfection at the interface. Therefore, we can speculate that the ion migration in the device may be impeded in accordance with the reduced *J*–*V* hysteresis.

### 2.4 Photophysical Properties

**Figure 5:**
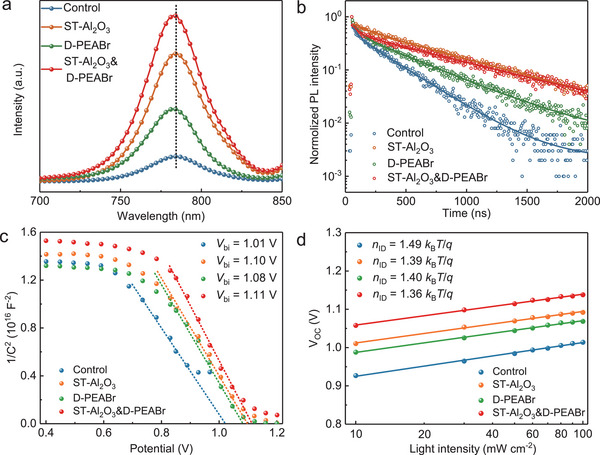
a) PL spectra and b) TRPL spectra of the perovskite films with different treatments. c) Mott–Schottky plots where the solid lines were fitted linearly and d) *V*_OC_ versus light intensity curves of the control, ST‐Al_2_O_3_, D‐PEABr, and ST‐Al_2_O_3_&D‐PEABr PSCs.

To further reveal the effect of surface treatments using Al_2_O_3_ nanoparticles and PEABr on charge recombination and transport properties, we performed the photoluminescence (PL) and time‐resolved PL (TRPL) spectroscopies for the control, ST‐Al_2_O_3_, D‐PEABr, and ST‐Al_2_O_3_&D‐PEABr perovskite films. The PL spectra of perovskite film treated with PEABr show a slight blue shift, which could be ascribed to the introduction of the Br element, slightly increasing the bandgap (**Figure** **5**a).^[^ ^41^ ^]^ The PL intensity of ST‐Al_2_O_3_&D‐PEABr film is substantially enhanced. Figure 5b displays the TRPL spectra fitted by the biexponential decay model to obtain the fast (*τ*_1_) and slow (*τ*_2_) decay lifetime related to trap‐assisted recombination at the surface and bulk, respectively.^[^ ^42^ ^]^ The average carrier lifetime (*τ*_ave_) can be determined to be 217.08, 643.93, 362.12, and 666.55 ns for the control, ST‐Al_2_O_3_, D‐PEABr, and ST‐Al_2_O_3_&D‐PEABr perovskite films, respectively. The improved PL intensity and longer carrier lifetimes of treated perovskite films than the control can be ascribed to the larger grain size and improved film quality, demonstrating reduced defect densities and suppressed trap‐assisted recombination. To shed light on this, the space‐charge‐limited current (SCLC) measurement of hole‐only devices was conducted to evaluate the trap‐state density (*n*_t_) of perovskite with or without surface modifications, as shown in Figure S16a–d, Supporting Information. The *n*_t_ is estimated from the formula shown in Supporting Information.^[^ ^43^ ^]^ The *V*_TFL_ is 0.712, 0.457, 0.627, and 0.341 V for the control, ST‐Al_2_O_3_, D‐PEABr, and ST‐Al_2_O_3_&D‐PEABr, respectively, corresponding to the *n*_t_ of 1.25 × 10^16^, 8 × 10^15^, 1.10 × 10^16^, and 5.97 × 10^15^ cm^−3^. The reduction of *n*_t_ can be explained by the improved crystallization and passivated defects by Al_2_O_3_ nanoparticles and PEABr, resulting in a substantial increase in *V*_OC_ and FF for the modified PSCs which is consistent with the above discussions. As normally acknowledge,^[^ ^44^ ^]^ the *V*_OC_ loss originating from non‐radiative recombination decreases with increasing the electroluminescence (EL) efficiency (EQE_EL_). Figure S17a, Supporting Information, shows the EQE_EL_ as a function of applied voltage curves of ST‐Al_2_O_3_, D‐PEABr, and ST‐Al_2_O_3_&D‐PEABr PSCs. It is noted that the control device could not operate as light‐emitting diodes (LEDs), so the EQE_EL_ of the control is not presented here. Clearly, the ST‐Al_2_O_3_&D‐PEABr device exhibits the highest EQE_EL_ with respect to other devices, indicative of the minimal *V*_OC_ loss, further illustrating the positive impact of Al_2_O_3_ nanoparticles and PEABr on suppressing the non‐radiative recombination via modulating the morphology and passivating defects at both buried and top interfaces of perovskite film.

In addition, electrochemical impedance spectroscopy (EIS) was conducted to collect the recombination resistance (*R*_rec_). Figure S17b, Supporting Information, presents the Nyquist plots of the control and other modified devices at an applied voltage of 0.85 V under dark conditions. Clearly, the *R*_rec_ of devices with modification is dramatically increased, indicating the significantly retarded charge recombination. To provide a deep insight into the carrier dynamic of PSCs, we performed the Mott–Schottky measurement to extract the built‐in potential (*V*_bi_). The capacitance–voltage (*C*–*V*) curves are provided in Figure 5c, and the *V*_bi_ can be deduced from the crossing with the *x*‐axis. Clearly, the *V*_bi_ remarkably increases (1.10, 1.08, and 1.11 V for ST‐Al_2_O_3_, D‐PEABr, and ST‐Al_2_O_3_&D‐PEABr, respectively) compared to the control (1.01 V), suggesting that the dual modification method is conducive to separate, transport, and extract the photogenerated charge carriers.^[^ ^45^ ^]^ Figure 5d shows the plots of *V*_OC_ as a function of light intensity, and the ideal factor (*n*_ID_) can be evaluated from the equation described in supporting information.^[^ ^37c^ ^]^ The *n*_ID_ values greater than and closer to 1 represent the appearance of defect‐assisted recombination and reduced defect‐assisted recombination in the device, respectively.^[^ ^46^ ^]^ It is found that the *n*_ID_ values show a clear decrease after modifications of Al_2_O_3_ nanoparticles and PEABr, proving that the modified devices have fewer defects, leading to lower non‐radiative recombination and improved *V*_OC_.

**Figure 6:**
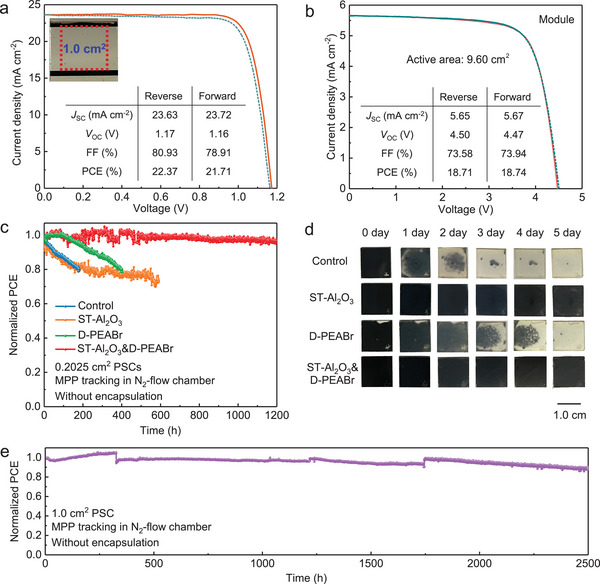
a) *J*–*V* curves of ST‐Al_2_O_3_&D‐PEABr devices with an area of 1 cm^2^. b) *J*–*V* curves of 5 cm × 5 cm module based on ST‐Al_2_O_3_&D‐PEABr structure with an active area of 9.60 cm^2^. c) The operational stability test of unencapsulated devices with an area of 0.2025 cm^2^ under one‐sun illumination at 25 °C. d) Optical images of the control, ST‐Al_2_O_3_, D‐PEABr, and ST‐Al_2_O_3_&D‐PEABr films aging under high humidity of 98 ± 1% within 5 days. e) The operational stability test of the unencapsulated device with area of 1 cm^2^ under one‐sun illumination at 25 °C.

To verify the universality of the Al_2_O_3_ nanoparticles and PEABr in different compositions, Cs_0.15_FA_0.85_PbI_3_ and Cs_0.05_FA_0.80_MA_0.15_PbI_2.5_Br_0.5_ perovskite systems were prepared, as shown in Figure S18, Supporting Information. The PV parameters are provided in Tables S2 and S3, Supporting Information. Obviously, for the Cs_0.15_FA_0.85_PbI_3_ system, the *V*_OC_ is extensively improved to 1.13 V for ST‐Al_2_O_3_&D‐PEABr device, compared with that of control with the *V*_OC_ of 0.93 V, and the *V*_OC_ could be increased to 1.16 (ST‐Al_2_O_3_&D‐PEABr) from 0.98 V (control) for Cs_0.05_FA_0.80_MA_0.15_PbI_2.5_Br_0.5_ perovskite system. These observations manifest that the dual modification strategy could be easily adopted in other perovskite systems to repress the notorious charge recombination and improve performance. In addition, we fabricated the 1 cm^2^ perovskite device based on the structure of ST‐Al_2_O_3_&D‐PEABr and Cs_0.15_FA_0.85_Pb(I_0.95_Br_0.05_)_3_ composition to validate the potential of these modifications in the scalable fabrication of PSCs, and the *J*–*V* curves are shown in **Figure** **6**a. A high PCE of 22.37% (*V*_OC_ = 1.17 V, *J*_SC_ = 23.63 mA cm^−2^, FF = 80.93%) is achieved. Furthermore, the large‐area module with the structure of ST‐Al_2_O_3_&D‐PEABr based on 5 cm × 5 cm substrate was fabricated through the spin coating and laser scribing methods, and the structure of the module is shown in Figure S19a, Supporting Information. As you can see from the *J*–*V* curves of the module (Figure 6b), the module exhibits a PCE of 18.71% with an active area of 9.60 cm^2^ (*V*_OC_ = 4.47 V, *J*_SC_ = 5.67 mA cm^−2^, FF = 73.94%), and the optical image of the module and laser patterning of P1, P2, and P3 lines are shown in Figure S19b, Supporting Information. These results pointed out that the modification strategy of Al_2_O_3_ nanoparticles and PEABr at both buried and top interfaces of perovskite films can not only be adequate in different perovskite composition systems but also be expanded to large‐area PSCs and modules, indicating the outstanding compatibility in realizing high‐performance PSCs.

### 2.5 Device Stability

As we know, stability is an important aspect to assess the possibility of commercialization for PSCs. The unencapsulated PSCs were kept in the N_2_ flowed box at the temperature of 25 °C. As shown in Figure 6c, the PCE of the control device decreased to 80% of the initial PCE after operating for 200 h, while the devices of ST‐Al_2_O_3_ and D‐PEABr can maintain for 600 and 400 h before the PCE decreased to 80% of the original value, respectively. It is worth mentioning that the ST‐Al_2_O_3_&D‐PEABr device exhibits 96% of the initial PCE after operating for 1200 h, indicative of much better operational stability than that of control. Moreover, we also measured the humidity stability by aging perovskite films at a high humidity condition of 98 ± 1%, as displayed in Figure 6d. It can be seen intuitively that the color of the control perovskite film is black after preparation, but it completely faded to light yellow within 5 days. For D‐PEABr perovskite film, the rate of color change is lower than that of control, but the black part also disappears on the fifth day. However, the color of perovskite films with the modification of Al_2_O_3_ at the buried interface hardly changes within 5 days, demonstrating that the introduction of Al_2_O_3_ nanoparticles could dramatically hamper the penetration of moisture and other external chemicals through the perovskite undersurface. The improved humidity stability may be attributed to the improved perovskite quality since the embedding of Al_2_O_3_ nanoparticles into the perovskite film induces the formation of compact morphology at the buried interface. Most astonishingly, the device with an area of 1 cm^2^ displays remarkable operational stability, as shown in Figure 6e, where 89% of its initial PCE could remain after 2500 h illumination. Furthermore, for the sub‐modules, the performance showed neglected degradation after encapsulation and storing in an N_2_‐filled glovebox for more than 1150 h (Figure S20, Supporting Information). The improved stability could be attributed to the collaborative effects of Al_2_O_3_ nanoparticles and PEABr. Specifically, the incorporation of Al_2_O_3_ nanoparticles could fill the voids at the buried interface and grain boundaries, reducing the dangling bonds at the interface, and thereby decreasing the defects, like ionic vacancies. Thus, the ion migration channels could be effectively reduced, meaning suppressed ion migration, as evidenced by reduced *J*–*V* hysteresis. Importantly, the improved carrier collection could inhibit the accumulation of charge carriers, impeding the phase segregation induced by the light or electric field.^[^ ^47^ ^]^ Moreover, the hydrophobic property of PEABr is expected to further prevent the ingression of moisture. In conclusion, the suppressed ion migration and phase segregation, dense morphology of buried interface, and the hydrophobic property of PEABr are considered to be the primary reasons for improved operational and moisture stabilities.

## 3 Conclusions

In summary, we propose an omnibearing strategy for the inverted PSCs to reduce the defect densities at the buried interface of perovskite film, as well as the top interface employing Al_2_O_3_ nanoparticles and ammonium salt PEABr. The Al_2_O_3_ nanoparticles were inserted into the buried interface of perovskite film to fill the voids between perovskite and HTL and reduce the dangling bonds at the interface, achieving the compact and flat morphology, as well as reduced imperfections at the buried interface. The PEABr introduced into the buried and top interface further improves the quality of perovskite film and passivates the interfacial defect states. Compared to the control film, the grain sizes of modified perovskite films increase, leading to fewer grain boundaries and recombination sites. These results indicate that the non‐radiative recombination is extensively repressed at both the buried and top interface, as well as in the bulk. As a result, the device of ST‐Al_2_O_3_&D‐PEABr realizes a dramatic enhancement in *V*_OC_ of 120 mV, compared to the control device (1.02 V), and the best PCE is as high as 23.1% with *V*_OC_ of 1.14 V, *J*_SC_ of 24.9 mA cm^−2^, and FF of 82%. Meanwhile, the ST‐Al_2_O_3_&D‐PEABr device with an area of 0.2025 cm^2^ (without mask) has excellent long‐term operational stability, maintaining 96% of its original PCE after operating for 1200 h. Most interestingly, the ST‐Al_2_O_3_&D‐PEABr perovskite film exhibits outstanding moisture stability. Moreover, this modification strategy could be easily expanded into other perovskite systems, large‐area solar cells, and modules. Particularly, the large‐area solar cell shows a PCE of 22.4% and excellent long‐term stability with 89% PCE of the initial value after MPP tracking for 2500 h. The improved performance could be mainly attributed to the suppression of non‐radiative recombination because of the morphology changes at the buried interface and reduced imperfections. Moreover, the suppressed ion migration owing to decreased defects and alleviated *J*–*V* hysteresis also account for improved operational stability. This work presents an important insight for modifying the buried interface using a dielectric layer of Al_2_O_3_ nanoparticles and passivating the perovskite surface using PEABr, which plays a significant role in manipulating the energy level alignment, perovskite film morphology, film quality, and suppression of trap‐assisted recombination.

## Conflict of Interest

The authors declare no conflict of interest.

## Supporting information

Supporting Information

## Data Availability

The data that support the findings of this study are available from the corresponding author upon reasonable request.
