# Supplementary material for: Buried Interface Dielectric Layer Engineering for Highly Efficient and Stable Inverted Perovskite Solar Cells and Modules
Source: Adv Sci (Weinh). 2023 Apr 25;10(19):2300586. doi: 10.1002/advs.202300586 (PMC10323608; doi:10.1002/advs.202300586)
Supplement: Supplementary file 1 — Supporting Information [file ADVS-10-2300586-s001.pdf]

ADVANCED SCIENCE
Open Access

Supporting Information

for *Adv. Sci.*, DOI 10.1002/advs.202300586

Buried Interface Dielectric Layer Engineering for Highly Efficient and Stable Inverted Perovskite Solar Cells and Modules

*Huan Li, Guanshui Xie, Xin Wang, Sibo Li, Dongxu Lin, Jun Fang, Daozeng Wang, Weixin Huang and Longbin Qiu**

Supporting Information

for

Buried Interface Dielectric Layer Engineering for Highly Efficient and Stable Inverted Perovskite Solar Cells and Modules

*Huan Li, Guanshui Xie, Sibo Li, Dongxu Lin, Xin Wang, Jun Fang, Daozeng Wang, Weixin Huang, and Longbin Qiu**

SUSTech Energy Institute for Carbon Neutrality, Department of Mechanical and Energy Engineering, Southern University of Science and Technology, Shenzhen, 518055, China

*Corresponding author: Longbin Qiu, Email: qiulb@sustech.edu.cn

Methods

Materials for perovskite cell preparation

Anhydrous dimethyl sulfoxide (DMSO), dimethylformamide (DMF) were purchased from Alfa Aesar. Isopropanol (IPA) was purchased from thermos scientific. Chlorobenzene (CB) was purchased form Sigma Aldrich. Ethyl alcohol, Ethyl acetate and Aluminum oxide nanoparticles (Al_2O_3 20 wt% in IPA, diameter 15-50 nm) was purchased from Aladdin. Lead iodine (PbI_2, 99.99%) and phenyl-C61-butyric acid methyl-ester ($PC_{61}BM$) were purchased from Advanced Election Technology. Formamidine iodide (FAI) was purchased from Greatcell Solar Materials. Cesium Bromide (CsBr) and [2-(3,6-dimethoxy-9H-carbazol-9-yl) ethyl] phosphonic acid (MeO-2PACz) were purchased from TCI. Phenethylammonium bromide (PEABr), fullerene (C_{60}) and bathocuproine (BCP) were purchased from Xi'an Polymer Light Technology. All chemicals were used as received.

Materials Preparations

Substrates preparation: The glass/indium tin oxide (ITO) substrates with the size of 1.5 cm × 1.5 cm were continuously cleaned in detergent water, distilled water and IPA using ultra-sonication for 30 min. In addition, all substrates were further treated with UV-ozone cleaner for 30 min before depositing the HTL.

Preparation of MeO-2PACz solution: The MeO-2PACz solution was dissolved in anhydrous ethyl alcohol with a concentration of 1 mg mL^{-1}. The obtained solution was put into a Vortex Shaker for 10 min before use.

Preparation of Al_2O_3 and PEABr solution: The Al_2O_3 dispersion solution was prepared by diluting 40 μL 20 wt% Al_2O_3 solution in 1.6 mL IPA. The Al_2O_3 dispersion solution was filtered using a 0.22 μm filter before the deposition. The PEABr solution was dissolved in DMF and IPA with the concentration of 1 mg mL^{-1}, respectively. For PEABr solution in IPA, 0.5 vol% DMF was added.

Preparation of perovskite solution: The $Cs_{0.15}FA_{0.85}Pb(I_{0.95}Br_{0.05})_3$ perovskite precursor solution (1.5 M) was used in this article. Specifically, the FAI (219.3 mg),

PbI_2 (691.5 mg), and CsBr (47.8 mg) were dissolved in 1 mL mixed solvent of DMF and DMSO with a volume ratio of 3:1. Then the precursor solution was vibrated overnight by using a Vortex Shaker. Note that the precursor solution was filtered using a 0.22 μm filter before the deposition.

Inverted solar cells and modules fabrication (*p*-i-*n*): For solar cells, the perovskite films were prepared through one step antisolvent method in an N_2-filled glovebox. The glass/ITO substrates were rapidly transferred to the N_2-filled glovebox after UV-ozone treatment. For control devices, 70 μL of MeO-2PACz (1 mg mL^{-1}) was dropped in the center of ITO substrates and spin-coated at 3000 rpm for 30 s after waiting for 15 s. After that, the substrates were kept at 100 °C for 10 min. For the devices with Al_2O_3 treatment, the diluted Al_2O_3 dispersion solution was spin-coated on the MeO-2PACz film at 5000 rpm for 30 s and heated at 100 °C for 10 min. For the passivation of buried surface using PEABr, the substrate was treated with 1 mg mL^{-1} PEABr solution dissolved in DMF through spin coating at 5000 rpm for 30 s and subsequently kept at 105 °C for 5 min on a hot plate. For deposition of perovskite film, the perovskite precursor solution was spin-coated on the ITO/MeO-2PACz, ITO/MeO-2PACz/Al_2O_3, ITO/MeO-2PACz/PEABr and ITO/MeO-2PACz/Al_2O_3/PEABr films at 5500 rpm for 55 s, respectively. During the spin-coating, 200 μL EA was immediately dropped in the center of spinning substrates at 10 s after starting the program. Then the perovskite films were annealed at 105 °C for 20 min. The devices with top surface passivation were fabricated by spin-coating PEABr solution dissolved in IPA on the top of perovskite film at 5000 rpm for 30 s, followed by keeping at a hot plate for 5 min with 70 °C. Hereafter, the PCBM solution with a concentration of 10 mg mL^{-1} in CB was deposited by 3000 rpm for 30 s. Finally, the 10 nm C_{60}, 8 nm BCP and 90 nm of metal electrode Ag were successively thermally evaporated with an evaporation rate of 0.1-1.0 Å s^{-1} at a pressure of around 4×10^{-4} Pa to obtain the complete devices. The photoactive area of the devices was 0.1 cm^2 and 1.0 cm^2. For a solar module, the fabrication is analogous to that of small-area solar cells except the amount of solution used in the preparing process, the size of glass/ITO substrate and laser scribing

patterning procedure. More dosage of solution is needed to completely cover the 5 cm × 5 cm substrate.

Material characterization

Grazing incidence X-ray diffraction (GIXRD) patterns were acquired in air by using a Rigaku Smartlab with Cu Kα radiation in the 2θ range of 3-60° at a scanning rate of 5° min^{-1}. The wavelength (λ) of incident X-ray is 1.5418 Å. The XPS was carried out on PHI 5000 Versaprobe III using Al Kα radiation (1486.7 eV) as an excitation source. UPS was conducted at the same equipment as XPS while using monochromatized He I radiation at 21.22 eV. The photoluminescence (PL) spectra and time-resolved PL (TRPL) were performed using an Edinburgh Instrument FLS1000 system applying a 450 nm laser as the excitation source. The scanning electronic microscope (SEM) images and energy dispersive X-ray spectroscopy mapping (EDX) were obtained from Apreo2 S Lovac field emission SEM at an acceleration voltage of 2-5 kV and a current of 25-50 pA. The contact angle measurements of the films were conducted on a KRUSS-The Drop Shape Analyzer DSA25 system and water was used as the wetting solvent. The roughness of the films was collected from the atomic force microscope (AFM) (Bruker, Dimension Edge). The transmittance and absorption of the films were attained from UV-vis spectroscopy (HITACHI, UH5700), employing an optical range from 300 to 1000 nm in air.

Solar cell Characterization

The current density versus voltage (*J-V*) characteristics of the PSCs were tested using a Keithley 2420 source meter under AM 1.5G one-sun illumination (100 $mW \cdot cm^{-2}$) which was produced by a solar simulator (Sol3A Class AAA, Oriel, Newport, USA) at room temperature. A standard reference silicon cell (91150-KG3, Newport, USA) was used to calibrate the light intensity. The metal masks with an area of 0.1 cm^2 and 1.0 cm^2 were employed to determine the active area of the PSCs. The incident photon to converted electron efficiency (IPCE) measurement was conducted to obtain the external quantum efficiency (EQE) spectra with a range from 300 to 850 nm using EQE system (IQE 200B, Newport). For the long-term stability measurement, a white light LED with

the intensity calibrated to satisfy one-sun conditions was used as the illumination source. The PSCs without encapsulation were put into a homemade box sealed and supplied with continuous N_2 flowing to control the relative humidity of ambient condition. The *J-V* curves were recorded by Multi-channel Solar Cell Stability Testing system (Ezhou Zhongneng Optoelectronics Co., Ltd.) every 20 min. The electroluminescence (EL) spectra were characterized by a Keithley 2420 source meter and integrating sphere connected to a spectrophotometer (QE65Pro). The Electrochemical impedance spectroscopy (EIS) and Mott-Schottky (M-S) curves of the devices were determined by using an Autolab electrochemical station with a frequency of 0.1 Hz to 1MHz in a dark environment.

For PL and TRPL measurements, the decay lifetime was obtained by fitting the formula of $f(t) = A_1 exp^{-t/\tau_1} + A_2 exp^{-t/\tau_2} + B$ using biexponential decay model. B, A_1, and A_2 are constants associated with baseline offset and the contributions of fast (τ_1) and slow (τ_2) segments, respectively. The average carrier lifetime (τ_{ave}) can be determined from the equation of $\tau_{ave} = \frac{\sum A_i \tau_i^2}{\sum A_i \tau_i}$.

For Space charge limited current (SCLC) measurements, the hole-only devices were scanned from 0 V to 1.2 V under dark condition, and the curves were recorded by a Keithley 2420 source meter. To estimate the trap density, n_t, the formula of $V_{TFL} = \frac{e n_t L^2}{2\varepsilon\varepsilon_0}$ was used, where V_{TFL}, e, L, ε, ε_0 are the trap-filled limit voltage, elementary charge (1.6 × 10^{-19} C), thickness of perovskite film (470 nm obtained from SEM pictures), relative dielectric constant (35), and vacuum permittivity (8.85 × 10^{-12} F m^{-1}), respectively.

For EL measurements, the voltage loss induced by the non-radiative recombination was calculated from the equation of $\Delta V_{OC}^{nonrad} = -\frac{k_B T}{q} \ln(EQE_{EL})$, where K_B, T, q, and EQE_{EL} are electron charge, Boltzmann constant, temperature and the electroluminescence efficiency.

For Mott-Schottky measurements, the relationship between the capacitance and voltage

is described by the M-S equation of $C^{-2} = \frac{2(V_{bi}-V)}{(A^2 q\varepsilon\varepsilon_0 N_A}$, where V, A, q, ε, ε_0, N_A parameters are applied voltage, active area of device, charge of electron, relative dielectric constant, vacuum permittivity and doping density of perovskite, respectively. Based on this formula, the built-in potential could be evaluated.

For the measurement of open-circuit voltage under different light intensities, the relationship between open-circuit voltage and light intensity could be described by the formula of $qV_{OC} = E_g + n_{ID}k_BTln\frac{I}{I_0}$, where q, k_B, T, I, E_g stand for electron charge, the Boltzmann constant, the absolute temperature, light intensity and optical bandgap of perovskite, respectively.

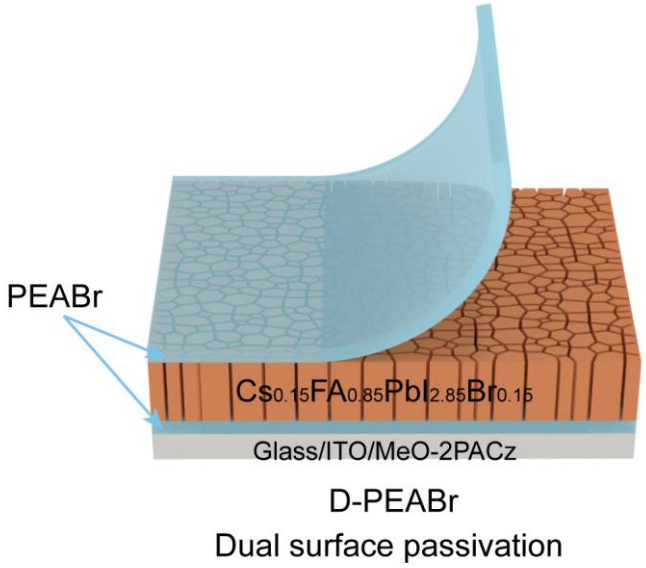

Figure S1. The schematic structure of surface passivation using PEABr on both top and buried interfaces of the perovskite absorber layer (D-PEABr).

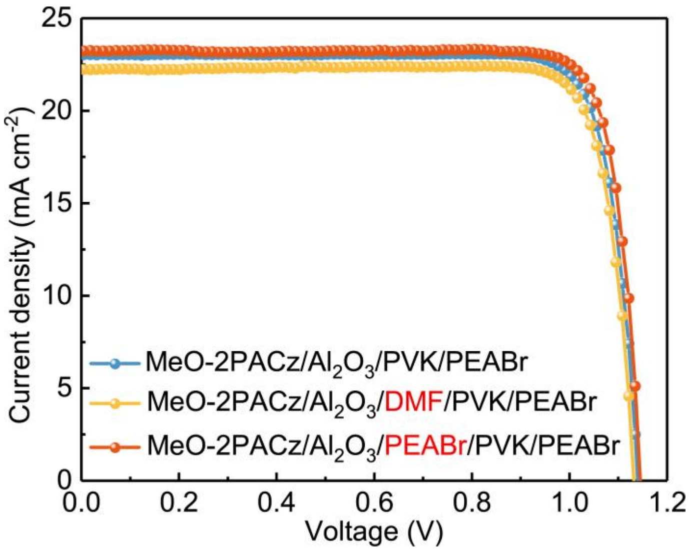

Figure S2. *J-V* curves of three different kinds of devices to show the importance of PEABr spin-coated on the bottom of perovskite.

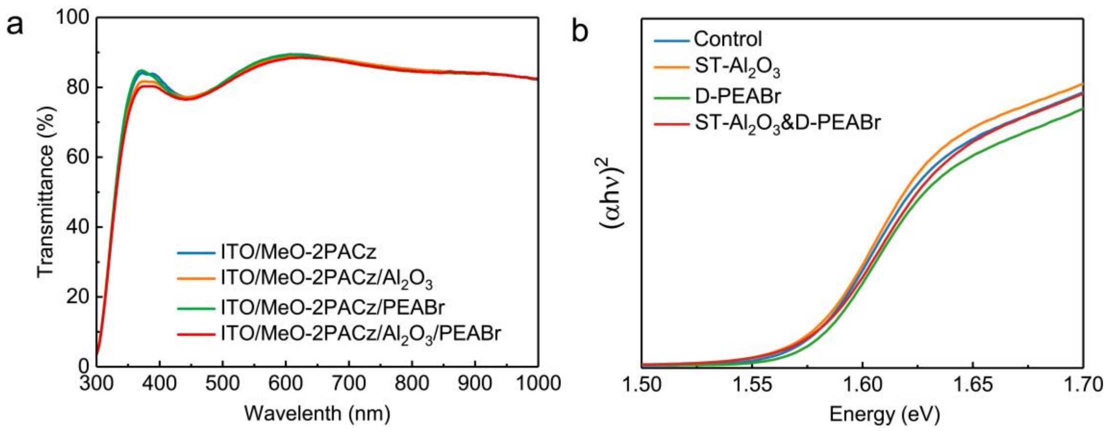

Figure S3. **(a)** The transmittance curves of the four different kinds of films. **(b)** The Tauc plots of the perovskite films with different treatments.

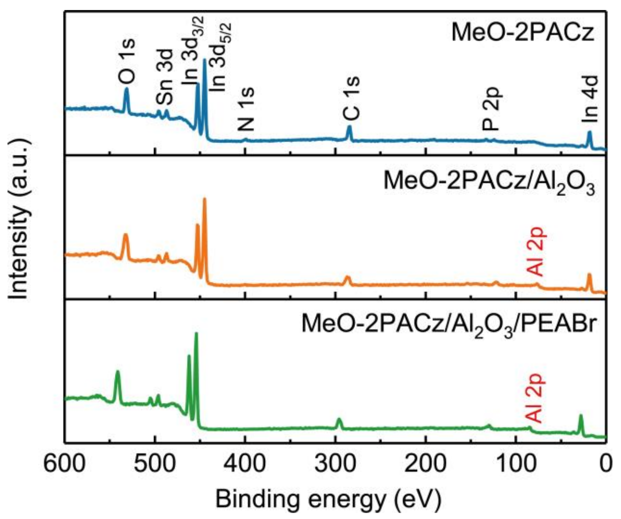

Figure S4. The XPS survey spectra of MeO-2PACz, MeO-2PACz/Al_2O_3 and MeO-2PACz/Al_2O_3/PEABr films.

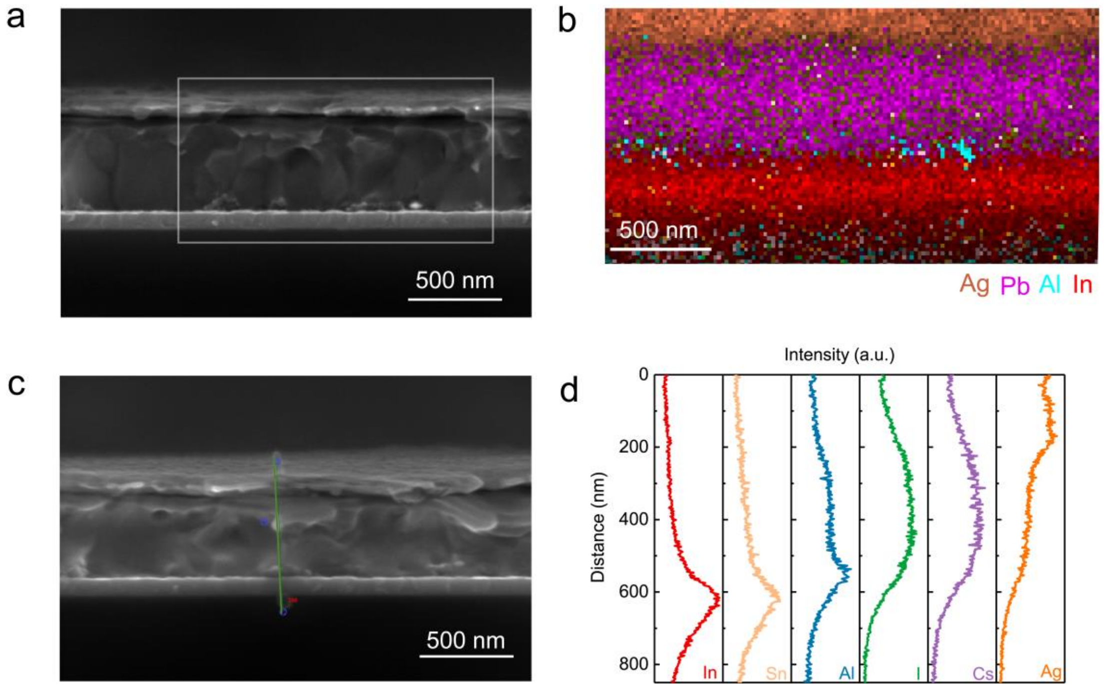

Figure S5. (a) Cross-section SEM image of ST-Al_2O_3&D-PEABr device and **(b)** corresponding EDX elemental mapping of Ag, Pb, Al and In. **(c)** Cross-section SEM image of ST-Al_2O_3&D-PEABr device and **(d)** corresponding EDX line scan.

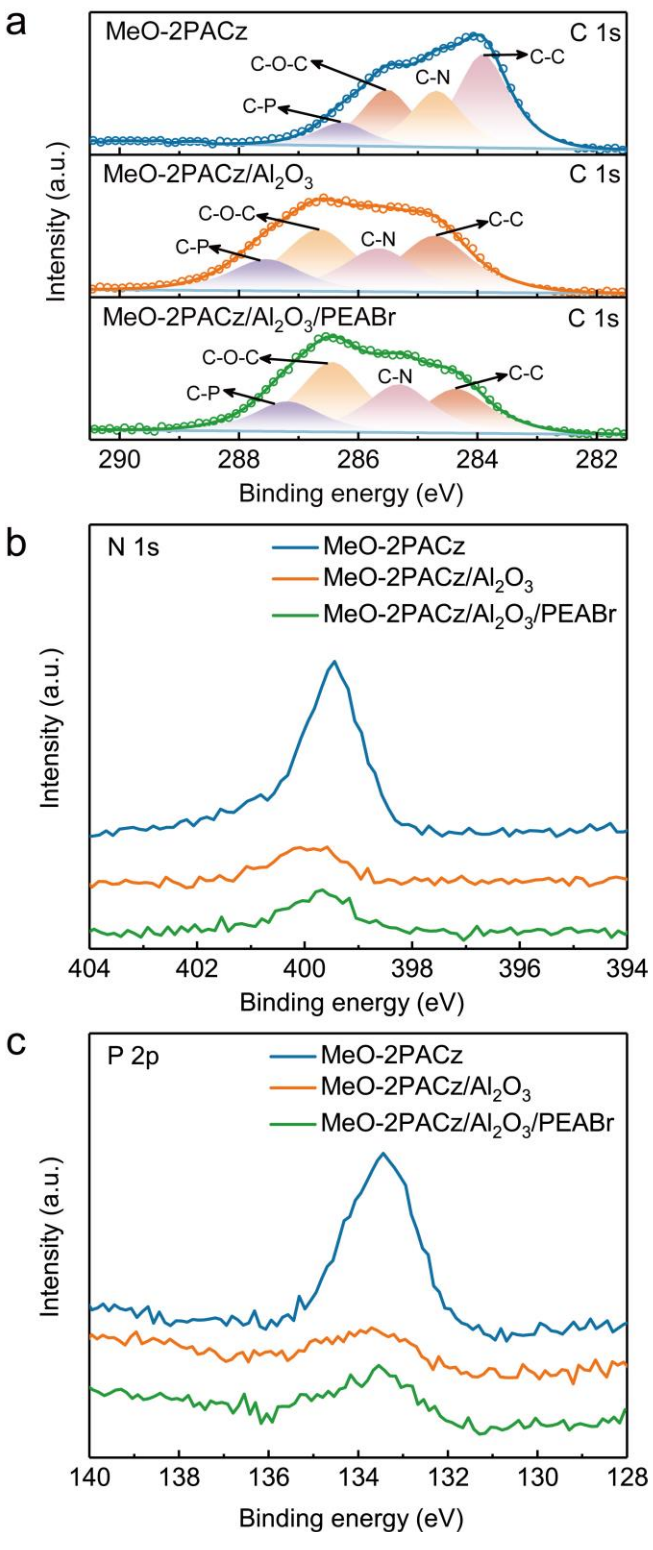

Figure S6. XPS spectra of the core levels of **(a)** C 1s, **(b)** N 1s and **(c)** P 2p of MeO-2PACz, MeO-2PACz/Al_2O_3 and MeO-2PACz/Al_2O_3/PEABr films.

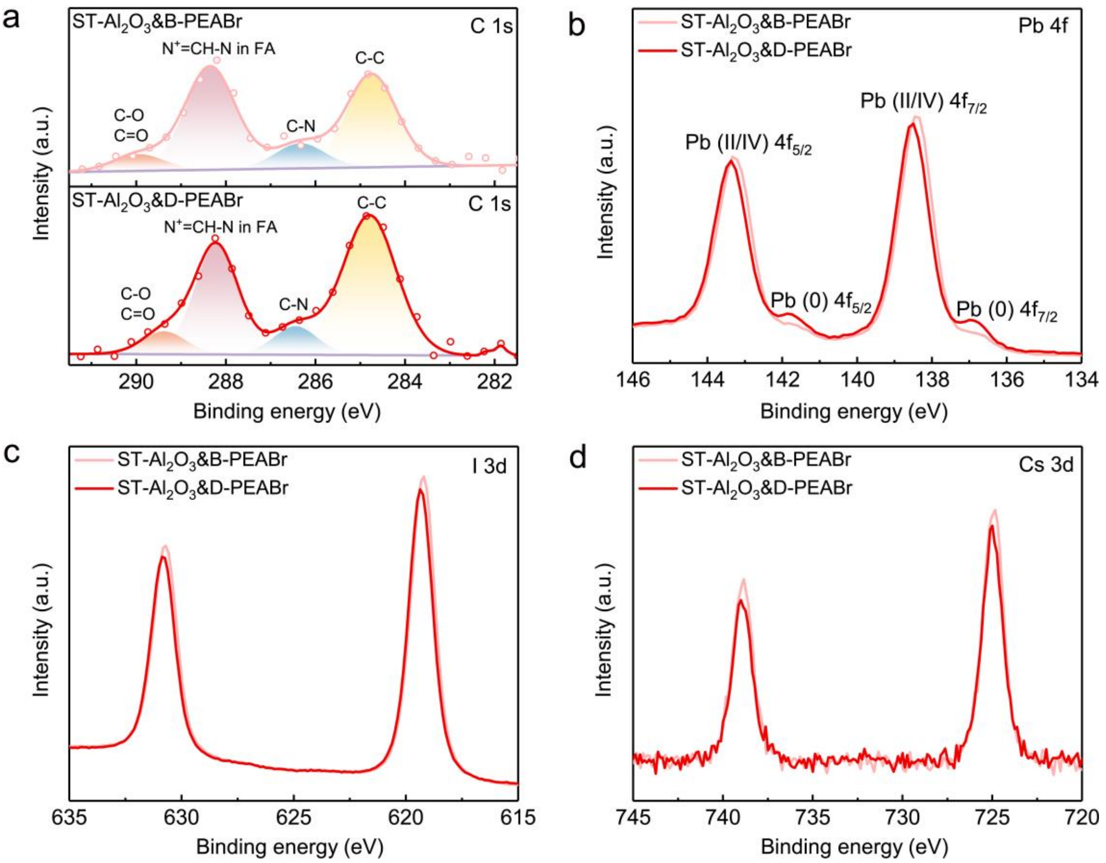

Figure S7. XPS spectra of **(a)** C 1s, **(b)** Pd 4f, **(c)** I 3d and **(d)** Cs 3d of ST-Al_2O_3&B-PEABr and ST-Al_2O_3&D-PEABr perovskite films.

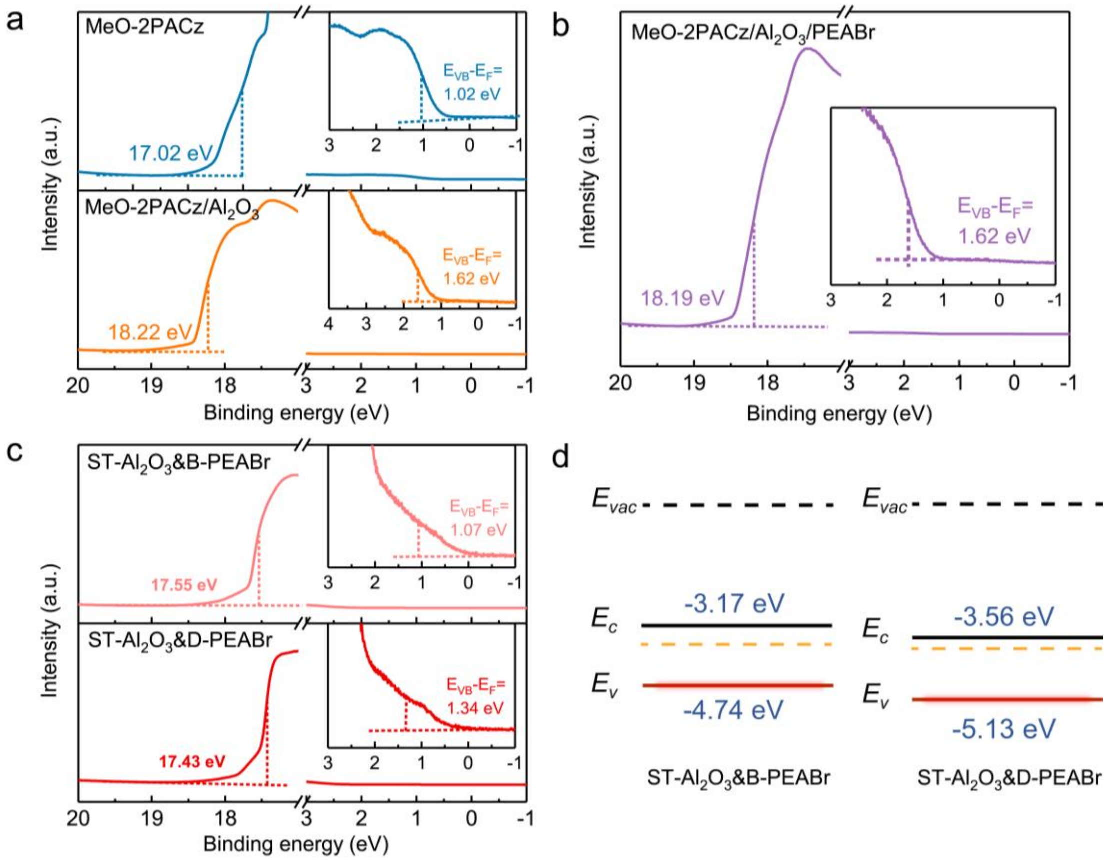

Figure S8. UPS spectra of **(a)** MeO-2PACz and MeO-2PACz/Al_2O_3, **(b)** MeO-2PACz/Al_2O_3/PEABr, **(c)** ST-Al_2O_3&B-PEABr and ST-Al_2O_3&D-PEABr films. The dash vertical lines of inset indicate the valence band maximum (E_V) with respect to the Fermi level. **(d)** Schematic energy level alignment diagram of ST-Al_2O_3&B-PEABr and ST-Al_2O_3&D-PEABr perovskite films. (E_c: conduction band minimum; E_{vac}: vacuum energy level)

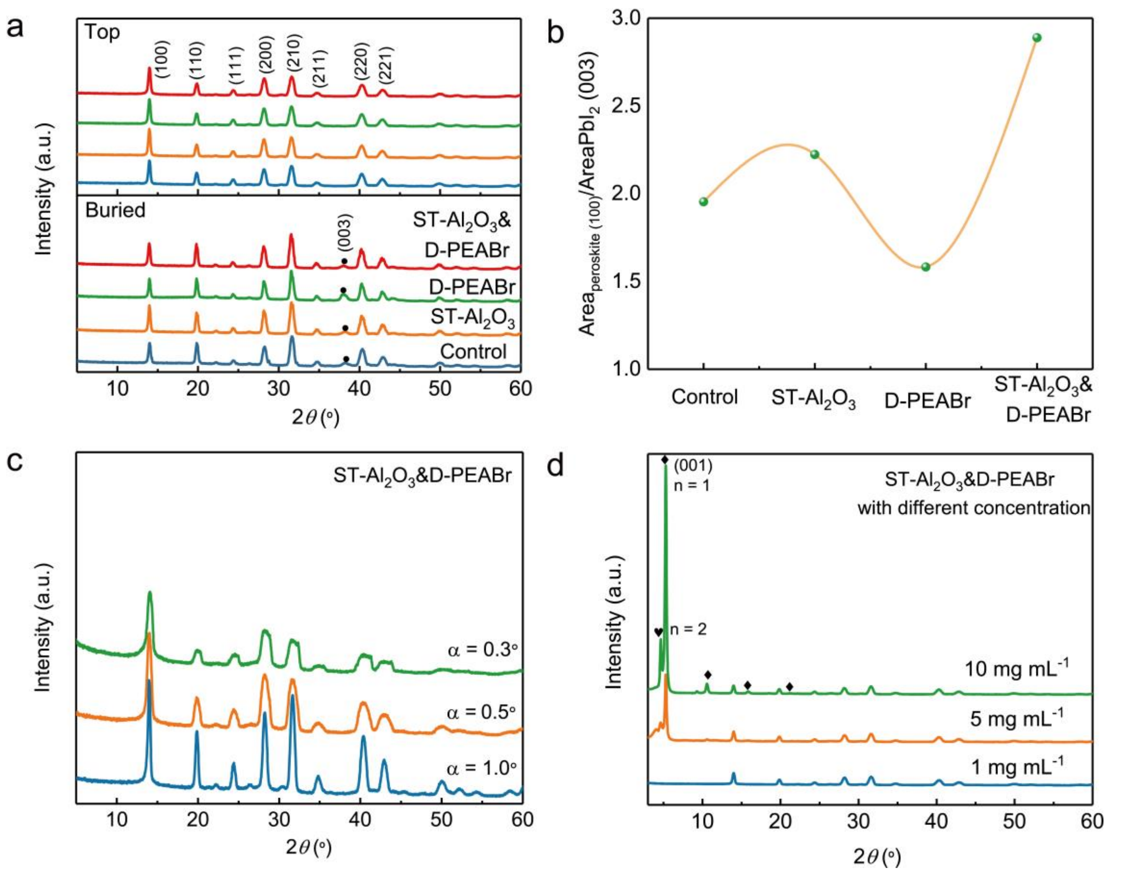

Figure S9. GIXRD patterns of **(a)** the top and buried interfaces of perovskite films for the control, ST-Al_2O_3, D-PEABr and ST-Al_2O_3&D-PEABr. The incident angle is 1.0°, where the solid circles denote the (003) diffraction peak of PbI_2. **(b)** The area ratio of perovskite (001) to PbI_2 (003). **(c)** The GIXRD patterns of ST-Al_2O_3&D-PEABr perovskite film (1 mg mL^{-1} PEABr on the top surface) with different incidence angle (α = 0.3°, 0.5°, 1°). **(d)** GIXRD patterns of ST-Al_2O_3&D-PEABr perovskite film with various concentration of PEABr on the top surface. The black diamonds and heat-shaped symbol denote the diffraction peaks resulting from 2D Ruddlesden-Popper phase.

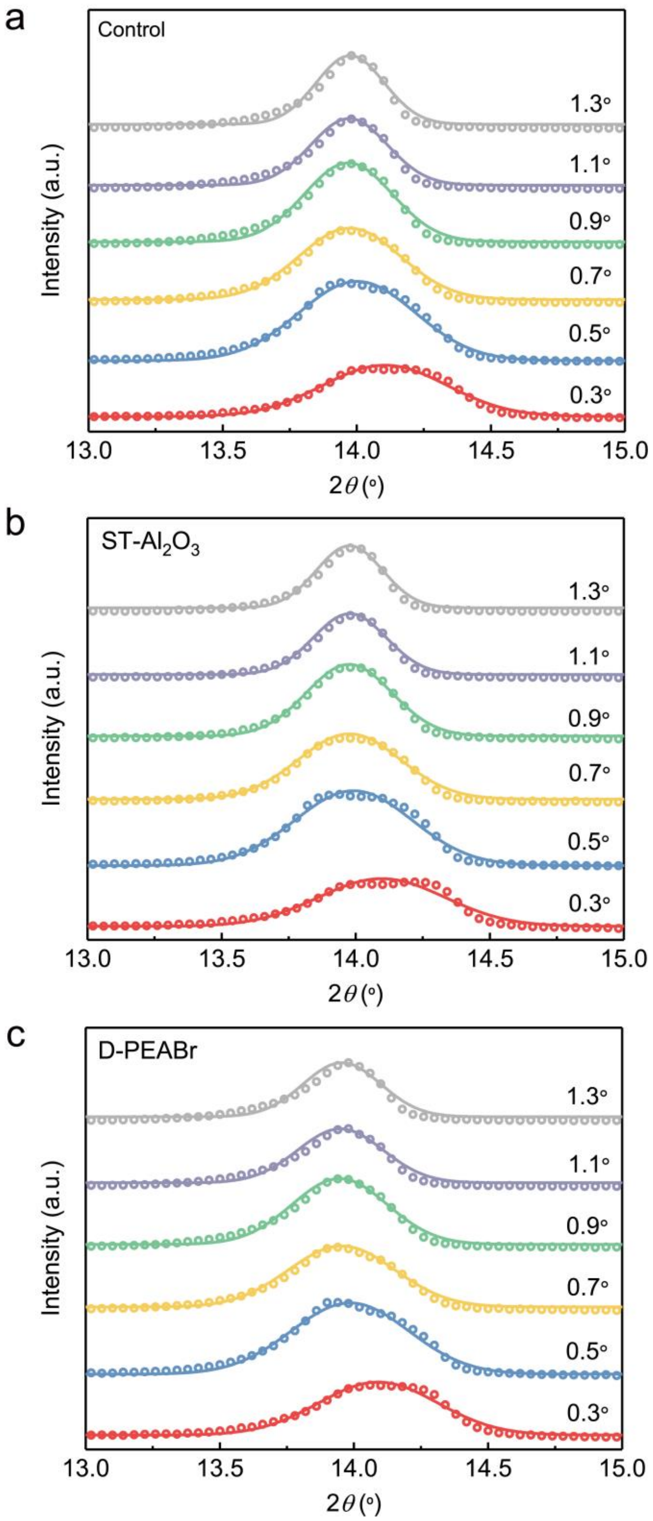

Figure S10. GIXRD patterns of **(a)** the control, **(b)** ST-Al_2O_3, and **(c)** D-PEABr perovskite films at the buried interface measured at different incidence angle (α).

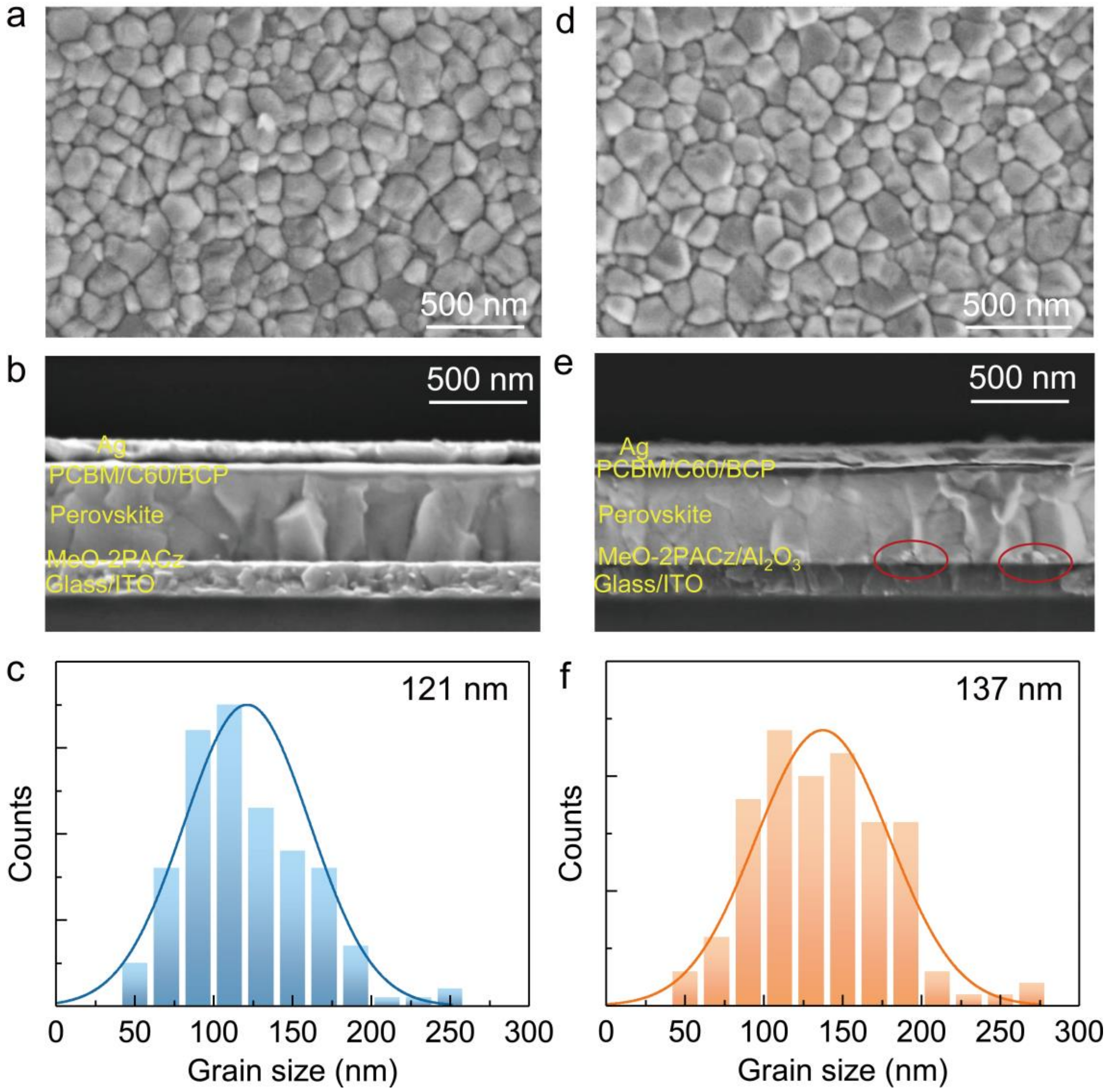

Figure S11. Top view SEM images, cross-section SEM images and the statistical distribution of crystal size of **(a)-(c)** the control and **(d)-(f)** ST-Al_2O_3 PSCs, respectively. The red circles in **(e)** denote the existence of Al_2O_3 nanoparticles.

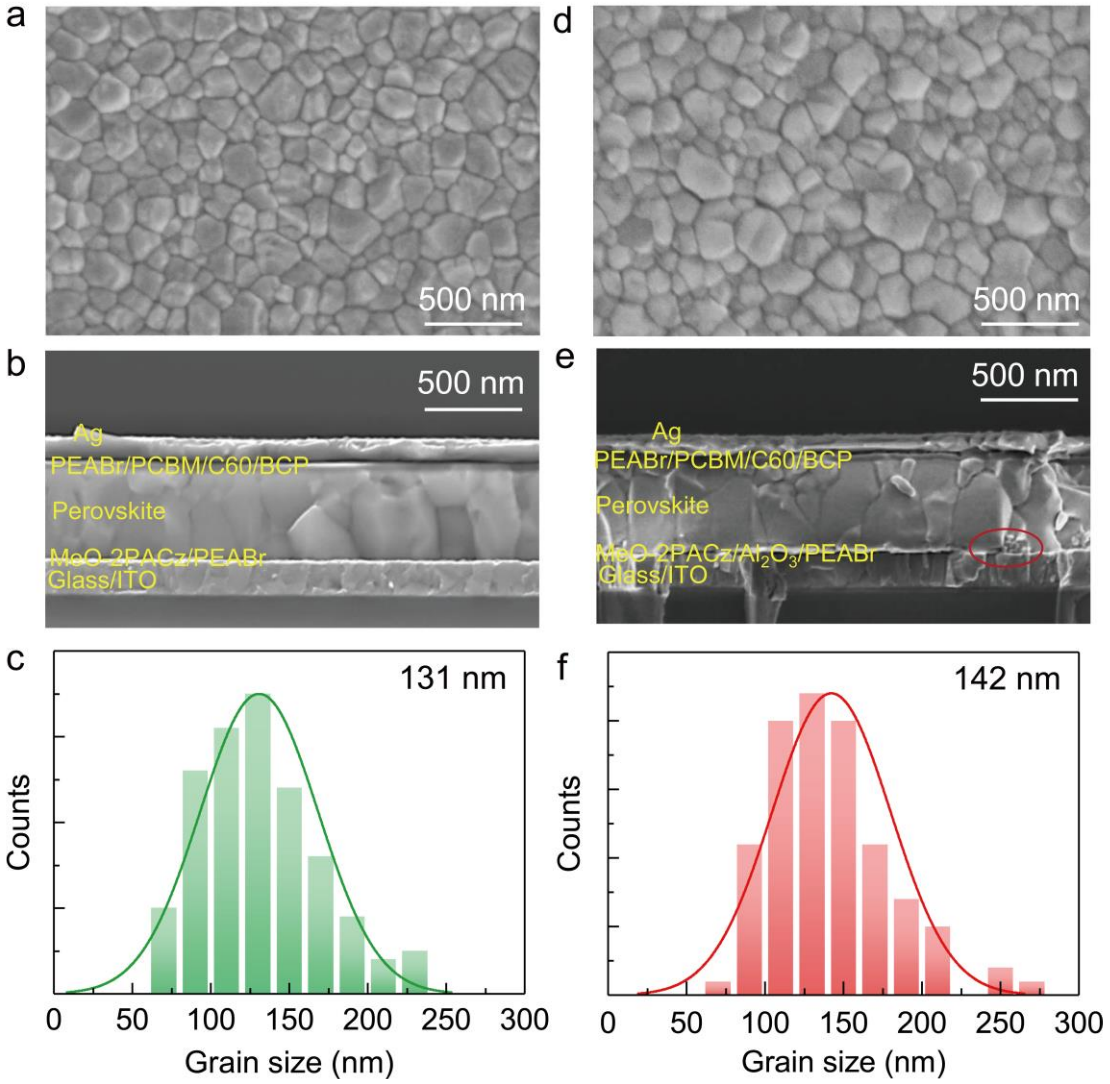

Figure S12. Top view SEM images, cross-section SEM images and the statistical distribution of crystal size of **(a)-(c)** D-PEABr and **(d)-(f)** ST-Al_2O_3&D-PEABr PSCs, respectively. The red circle in **(e)** denotes the existence of Al_2O_3 nanoparticles.

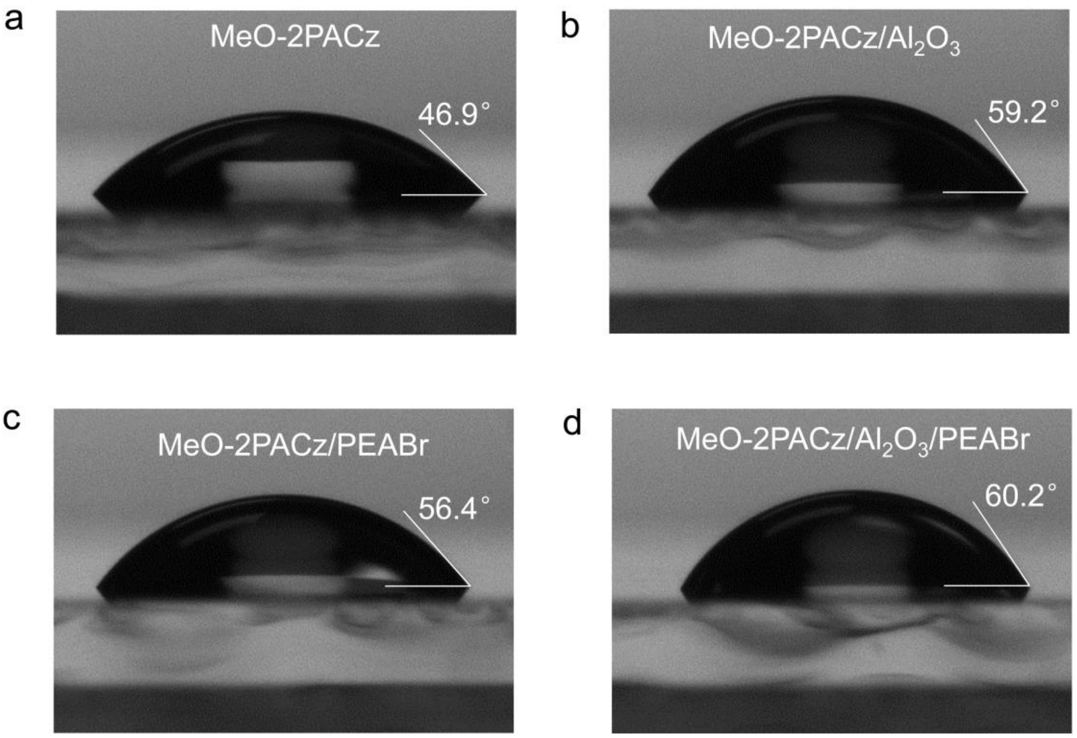

Figure S13. Water contact angle of **(a)** MeO-2PACz, **(b)** MeO-2PACz/Al_2O_3, **(c)** MeO-2PACz/PEABr and **(d)** MeO-2PACz/Al_2O_3/PEABr films on glass/ITO substrates.

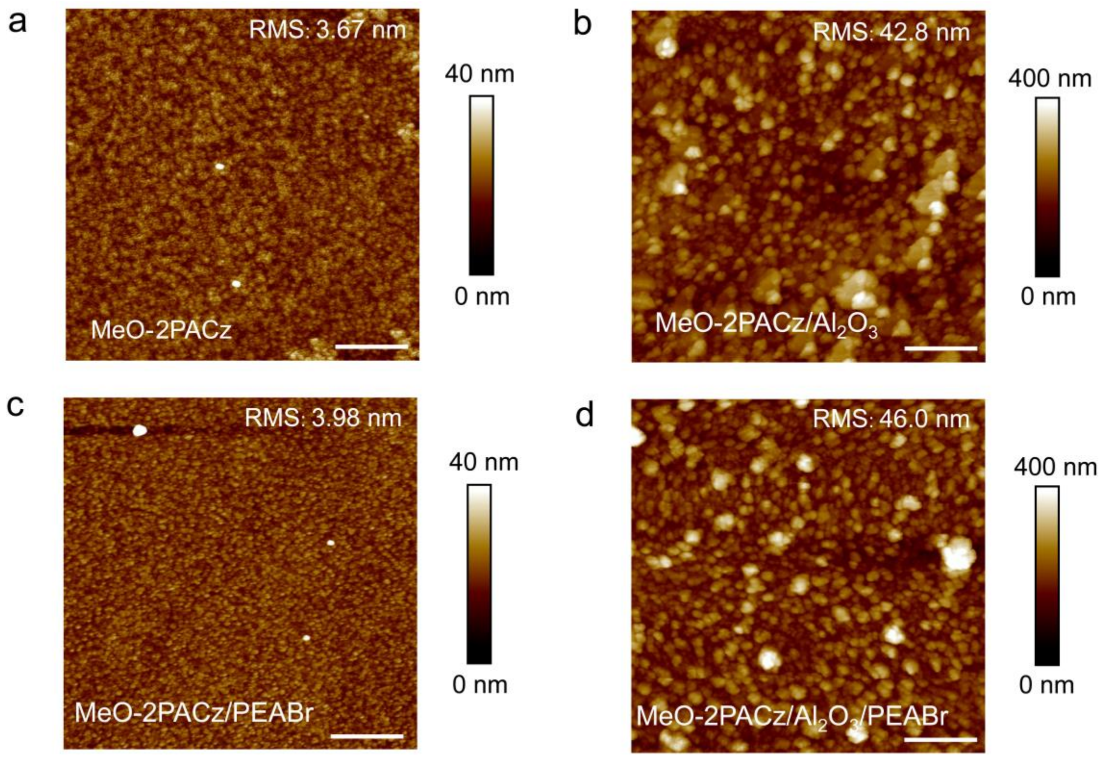

Figure S14. AFM topographic images of **(a)** MeO-2PACz, **(b)** MeO-2PACz/Al_2O_3, **(c)** MeO-2PACz/PEABr and **(d)** MeO-2PACz/Al_2O_3/PEABr films on glass/ITO substrates. The scale bar is 2 μm.

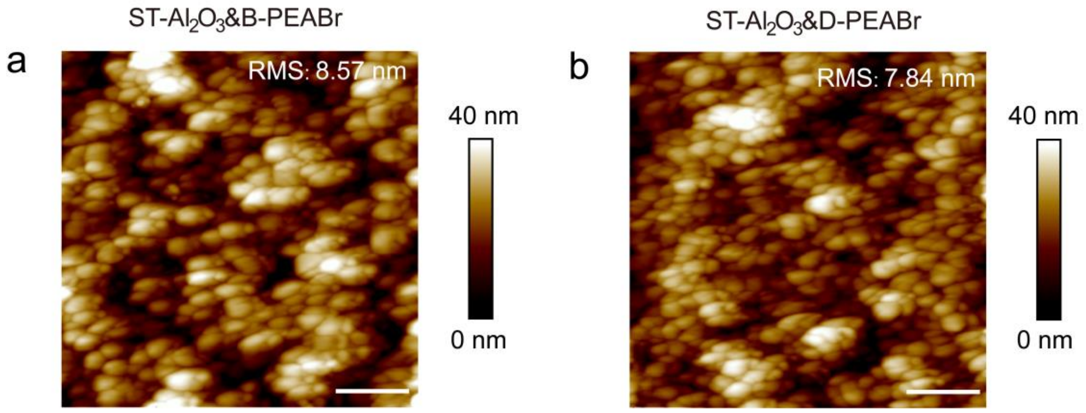

Figure S15. AFM topographic images of **(a)** ST-Al_2O_3&B-PEABr and **(b)** ST-Al_2O_3&D-PEABr perovskite films. The scale bar is 600 nm.

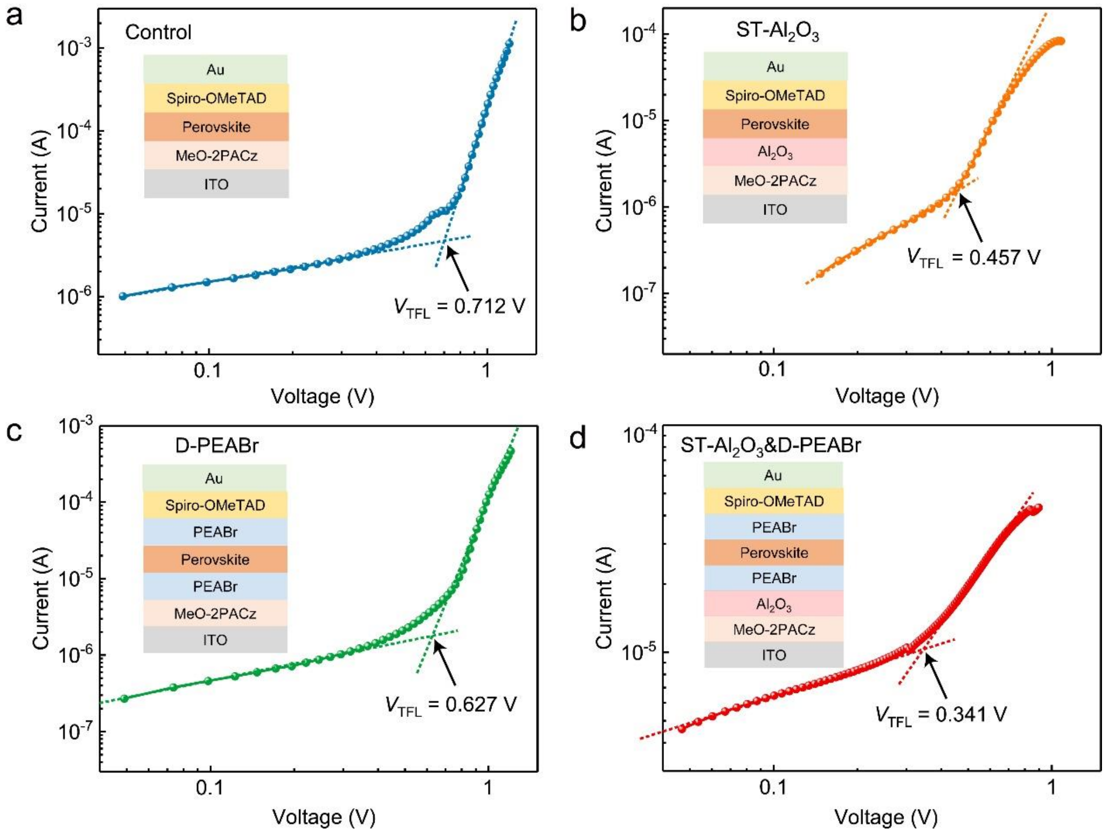

Figure S16. SCLC spectra of **(a)** the control, **(b)** ST-Al_2O_3, **(c)** D-PEABr and **(d)** ST-Al_2O_3&D-PEABr PSCs with a hole-only device structure.

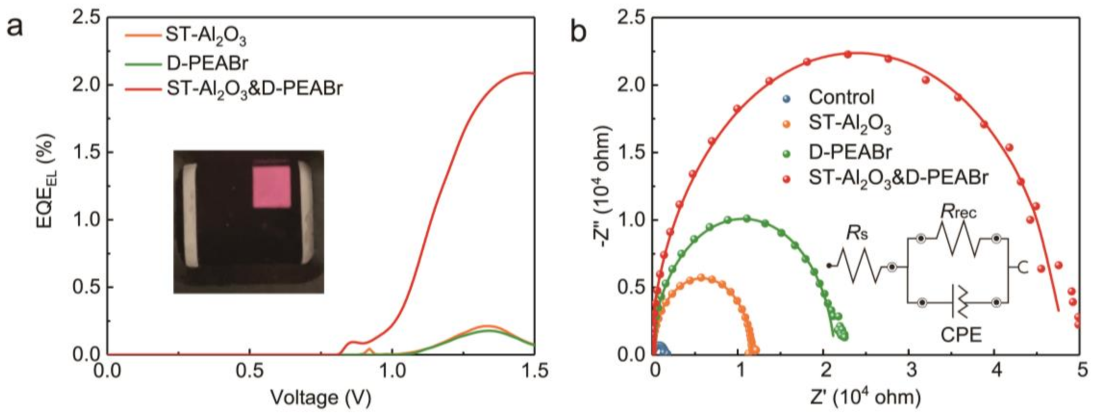

Figure S17. **(a)** The plots of EQE_{EL} versus voltage. The inset displays the best performing device of ST-Al_2O_3&D-PEABr operated as light-emitting diode (LED). **(b)** Nyquist plots (0.1 Hz to 1 MHz in the dark and applied voltage is 0.85 V) of the control, ST-Al_2O_3, D-PEABr and ST-Al_2O_3&D-PEABr.

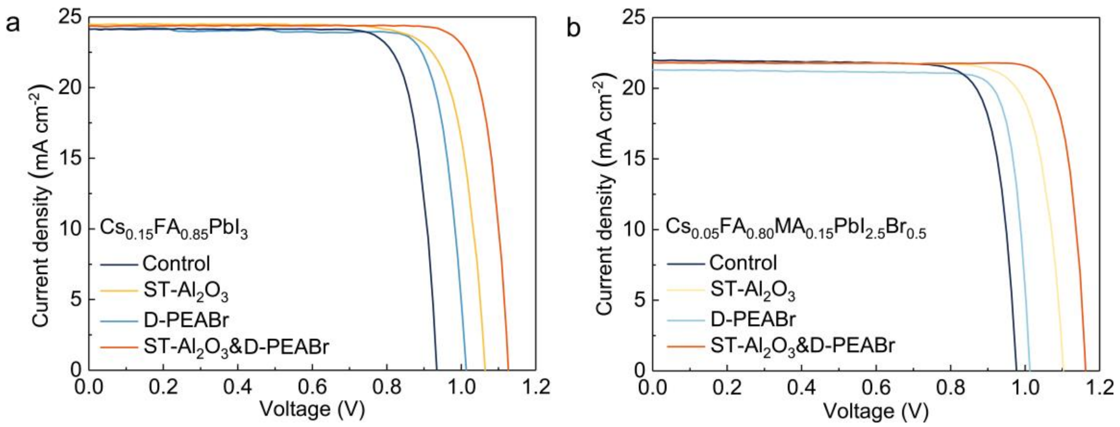

Figure S18. *J-V* curves of the control, ST-Al_2O_3, D-PEABr and ST-Al_2O_3&D-PEABr PSCs for **(a)** $Cs_{0.15}FA_{0.85}PbI_3$ and **(b)** $Cs_{0.05}FA_{0.80}MA_{0.15}PbI_3$ compositions.

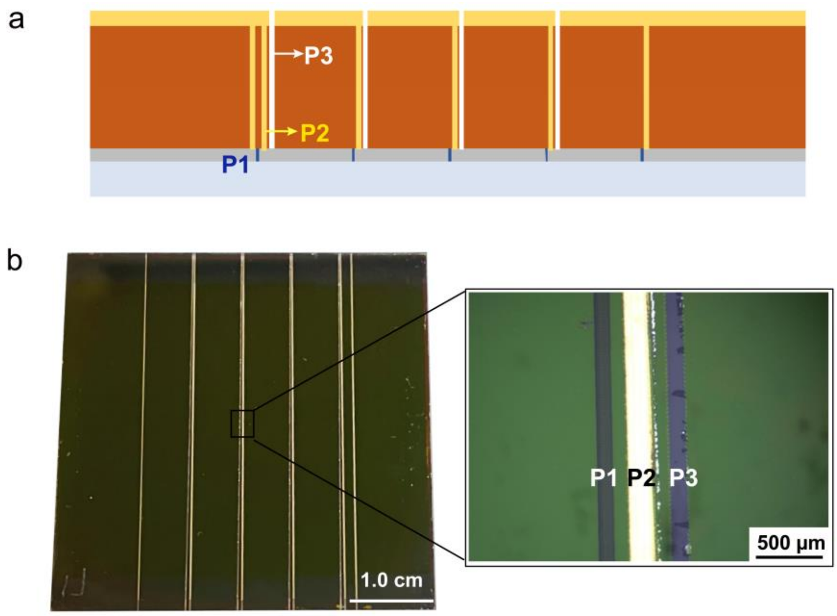

Figure S19. **(a)** Structure of the solar cell module. **(b)** Optical image of the module and laser patterning of P1, P2 and P3 lines.

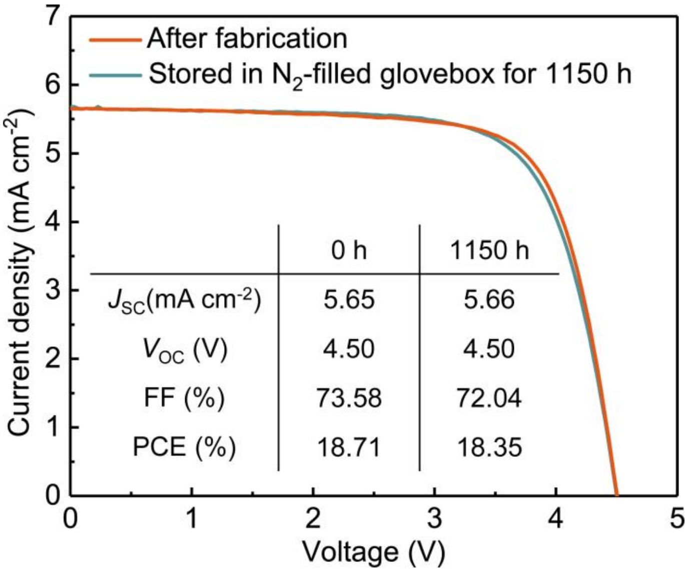

Figure S20. *J-V* curves of 5 cm × 5 cm sub-module based on ST-Al_2O_3&D-PEABr structure with an active area of 9.60 cm^2 measured after fabrication and storing in N_2-filled glovebox for 1150 h.

Table S1. Summary of MA-free high efficiency perovskite solar cells with inverted structure.

Composition	Highest efficiency	Area	Stability	Reference
$FA_{0.85}Cs_{0.15}PbI_3$	20.70%	0.04 cm^2	1,000 h 95% MPP	[S1]
$Cs_{0.05}FA_{0.95}PbI_3$	21.25%	0.1 cm^2	1,000 h 95% MPP	[S2]
$Cs_{0.17}FA_{0.83}Pb(I_{0.8}Br_{0.2})_3$	21.10%	0.108 cm^2	500 h 95% MPP	[S3]
$Cs_{0.15}FA_{0.85}Pb(I_{0.9}Br_{0.1})_3$	21.64%	0.09 cm^2	1,000 h storage at 85 °C	[S4]
$Cs_{0.18}FA_{0.82}PbI_3$	22.70%	0.12 mm^2	1,000 h storage at 85 °C	[S5]
$Cs_{0.25}FA_{0.75}Pb(I_{0.85}Br_{0.15})_3$	21.65%	0.06 cm^2	Not given	[S6]
$Cs_{0.13}FA_{0.87}Pb(I_{0.95}Br_{0.05})_3$	20.89%	0.059 cm^2	Not given	[S7]
$FAPbI_3$	22.13%	0.09 cm^2	800 h 92% aging at air	[S8]
$Cs_{0.05}FA_{0.95}PbI_3$	23.50%	0.09 cm^2	Not given	[S9]
$Cs_{0.05}FA_{0.95}PbI_3$	23.49%	0.08 cm^2	600 h 94% MPP	[S10]
$Cs_{0.03}FA_{0.97}PbI_3$	23.60%	0.04 cm^2	2,200 h 95% MPP	[S11]
$Cs_{0.15}FA_{0.85}Pb(I_{0.85}Br_{0.15})_3$	23.1%	0.1 cm^2	1,200 h 96 % MPP	This work
	22.4%	1.0 cm^2	2,500 h 89 % MPP	

Table S2. The champion photovoltaic parameters of perovskite solar cells based on the control, ST-Al_2O_3, D-PEABr and ST-Al_2O_3&D-PEABr. The perovskite composition is $Cs_{0.15}FA_{0.85}PbI_3$.

Device type	V_{OC} (V)	J_{SC} (mA cm^{-2})	FF (%)	PCE (%)
Control	0.93	24.12	81.81	18.43
ST-Al_2O_3	1.06	24.48	79.91	20.80
D-PEABr	1.01	24.15	83.10	20.32
ST-Al_2O_3&D-PEABr	1.13	24.35	84.38	23.10

Table S3. The champion photovoltaic parameters of perovskite solar cells based on the control, ST-Al_2O_3, D-PEABr and ST-Al_2O_3&D-PEABr. The perovskite composition is $Cs_{0.05}FA_{0.80}MA_{0.15}PbI_{2.5}Br_{0.5}$.

Device type	V_{OC} (V)	J_{SC} (mA cm^{-2})	FF (%)	PCE (%)
Control	0.98	21.96	81.90	17.54
ST-Al_2O_3	1.10	21.80	81.99	19.69
D-PEABr	1.01	21.31	85.45	18.40
ST-Al_2O_3&D-PEABr	1.16	21.80	86.22	21.80

Reference

[S1] Z. Peng, Q. Wei, H. Chen, Y. Liu, F. Wang, X. Jiang, W. Liu, W. Zhou, S. Ling, Z. Ning, *Cell Rep. Phys. Sci.* **2020**, 1, 100224.

[S2] D. Li, Y. Huang, G. Wang, Q. Lian, R. Shi, L. Zhang, X. Wang, F. Gao, W. Kong, B. Xu, C. Cheng, S. Li, *J. Mater. Chem. A* **2021**, 9, 12746-12754.

[S3] S. Li, K. Fan, Y. Cui, S. Leng, Y. Ying, W. Zou, Z. Liu, C.-Z. Li, K. Yao, H. Huang, *ACS Energy Lett.***2020**, 5, 2015-2022.

[S4] Y. Chen, W. Tang, Y. Wu, X. Yu, J. Yang, Q. Ma, S. Wang, J. Jiang, S. Zhang, W.-H. Zhang, *Chem. Eng. J.* **2021**, 425, 131499.

[S5] S. Gharibzadeh, P. Fassl, I. M. Hossain, P. Rohrbeck, M. Frericks, M. Schmidt, T. Duong, M. R. Khan, T. Abzieher, B. A. Nejand, F. Schackmar, O. Almora, T. Feeney, R. Singh, D. Fuchs, U. Lemmer, J. P. Hofmann, S. A. L. Weber, U. W. Paetzold, *Energy Environ. Sci.* **2021**, 14, 5875-5893.

[S6] K. M. W. Peng, F. Cai, H. Meng, Z. Zhu, T. Li, S. Yuan, X. Feng, J. Xu, M. D. McGehee, J. Xu, *Science* **2023**, 379, 683-690.

[S7] Q. Jiang, J. Tong, Y. Xian, R. A. Kerner, S. P. Dunfield, C. Xiao, R. A. Scheidt, D. Kuciauskas, X. Wang, M. P. Hautzinger, R. Tirawat, M. C. Beard, D. P. Fenning, J. J. Berry, B. W. Larson, Y. Yan, K. Zhu, *Nature* **2022**, 611, 278-283.

[S8] D. Zhang, H. Zhang, H. Guo, F. Ye, S. Liu, Y. Wu, *Adv. Funct. Mater.* **2022**, 32, 2200174.

[S9] X. Li,W. Zhang, X. Guo, C. Lu, J. Wei, J. Fang, *Science* **2022**, 375, 434-437.

[S10] M. Li, H. Li, Q. Zhuang, D. He, B. Liu, C. Chen, B. Zhang, T. Pauporté, Z. Zang, J. Chen, *Angew. Chem. Int. Ed.* **2022**, 61, e202206914.

[S11] T. Pan, W. Zhou, Q. Wei, Z. Peng, H. Wang, X. Jiang, Z. Zang, H. Li, D. Yu, Q. Zhou, M. Pan, W. Zhou, Z. Ning, *Adv. Mater.* **2023**, 2208522.
